# Mitigating Effect of *Trans*-Zeatin on Cadmium Toxicity in *Desmodesmus armatus*

**DOI:** 10.3390/cells13080686

**Published:** 2024-04-15

**Authors:** Alicja Piotrowska-Niczyporuk, Elżbieta Bonda-Ostaszewska, Andrzej Bajguz

**Affiliations:** 1 Department of Biology and Plant Ecology, Faculty of Biology, University of Bialystok, Ciolkowskiego 1J, 15-245 Bialystok, Poland; alicjap@uwb.edu.pl; 2 Department of Evolutionary and Physiological Ecology, Faculty of Biology, University of Bialystok, Ciolkowskiego 1J, 15-245 Bialystok, Poland; elabonda@uwb.edu.pl

**Keywords:** algae, antioxidants, cadmium, cytokinin, photosynthetic pigments, phytochelatins

## Abstract

Phytohormones, particularly cytokinin *trans*-zeatin (*t*Z), were studied for their impact on the green alga *Desmodesmus armatus* under cadmium (Cd) stress, focusing on growth, metal accumulation, and stress response mechanisms. Using atomic absorption spectroscopy for the Cd level and high-performance liquid chromatography for photosynthetic pigments and phytochelatins, along with spectrophotometry for antioxidants and liquid chromatography–mass spectrometry for phytohormones, we found that *t*Z enhances Cd uptake in *D. armatus*, potentially improving phycoremediation of aquatic environments. Cytokinin mitigates Cd toxicity by regulating internal phytohormone levels and activating metal tolerance pathways, increasing phytochelatin synthase activity and phytochelatin accumulation essential for Cd sequestration. Treatment with *t*Z and Cd also resulted in increased cell proliferation, photosynthetic pigment and antioxidant levels, and antioxidant enzyme activities, reducing oxidative stress. This suggests that cytokinin-mediated mechanisms in *D. armatus* enhance its capacity for Cd uptake and tolerance, offering promising avenues for more effective aquatic phycoremediation techniques.

## 1. Introduction

Cadmium (Cd) is one of the most toxic heavy metals, and its pollution has become a global issue for environmental sustainability [1]. The presence of Cd in natural waters is usually less than 1 μg L^−1^, but the values can vary greatly among different localities [2]. Similar to other heavy metals, Cd can be easily taken up by phytoplankton from the aquatic environment. The accumulation of Cd by green algae not only affects the growth and development of aquatic organisms but may also enter the food chain and endanger human health [3]. Studying the algal biochemical response to Cd stress conditions and resistance mechanisms can provide a reference for the better application of algae in bioremediation. The rapid reduction in the levels of metals in water after microalgal treatment demonstrates high microalgal efficiency for the removal of metals according to the requirements of international standards [4,5,6,7,8]. Green algae are among the most promising organisms for heavy metal biosorption due to their simple nutritional needs, higher surface area relative to volume, and high growth ratio. Metals removed by this group of aquatic plants can be easily recovered from biomass and recycled [9,10]. Hence, the algal bioremediation approach, known as phycoremediation, is considered an economical and eco-friendly technique for water treatment [11].

The growth of microalgae can serve as an indicator of water pollution since they typically respond to metal ions, such as Cd [4,12]. Moreover, the growth inhibition induced by heavy metals may be associated with disturbances in phytohormone homeostasis. For example, lead (Pb) stress stimulates the synthesis of abscisic acid (ABA) and reduces the levels of auxins, brassinosteroids, cytokinins, and gibberellins involved in cell proliferation. Thus, a lower growth ratio was observed in the green alga *Acutodesmus obliquus* treated with Pb [13,14]. Additionally, oxidative stress in living organisms can be connected with increased concentration and the toxicity of metals. Thus, an increase in the level of reactive oxygen species (ROS), an enhanced lipid peroxidation process, and/or a reduction in cellular antioxidant capacity, i.e., enzymatic (ascorbate peroxidase, APX; glutathione reductase, GR; catalase, CAT; superoxide dismutase, SOD) and non-enzymatic (ascorbate; glutathione, GSH; proline, Pro) antioxidants, is often observed [15]. Oxidative stress can be associated with the inhibition of photosynthesis, chlorophyll synthesis, and changes in the content of other photosynthetic pigments, such as carotenes and xanthophylls [15,16].

Vascular plants and algae have adopted several strategies to mitigate the toxic effects of Cd. The primary detoxification mechanism involves the production of phytochelatins (PCs), which are engaged in sequestration of metal ions into biologically inactive forms [17,18]. PCs are low-molecular-weight thiols with the general structure: (γ-Glu-Cys)_n_-Gly (n = 2–11). PC_2–5_ are the predominant peptides found in phytoplankton. Phytochelatin synthase (PCS) is an enzyme involved in PCs’ synthesis from precursor tripeptide glutathione (GSH) [19]. PCs can sequester metals by chelating them with the sulfhydryl groups present in cysteines (Cys), building PC peptides [20,21,22,23].

Reducing the toxicity of heavy metals remains a major challenge for all developing countries. Recently, studies have also explored the treatment of plants and algae with exogenous phytohormones to increase stress tolerance for phytoremediation purposes [13,14,24,25,26,27]. Phytohormones are active biological molecules that play a crucial role in triggering plants’ responses to abiotic stress. Over the past decade, significant efforts have been made to understand the roles of auxins, brassinosteroids, cytokinins, gibberellins, and ABA in the acclimatization of green algae and vascular plants to metal stress [28,29,30]. For instance, phytohormones, including indole-3-acetic acid (IAA), brassinolide (BL), and ABA, have improved the *Sedum alfredii* phytoextraction potential and positively affected plant growth and the antioxidant system in the presence of heavy metals, such as Cd, Pb, and zinc (Zn) [31]. Other studies have also confirmed that cytokinins, a group of plant hormones extensively involved in the regulation of plant growth and development, play a key role in plant adaptation to heavy metal stress and improve plant phytoremediation properties [26,32].

The level of active cytokinins decreases under the influence of heavy metals in vascular plants, e.g., in *Triticum durum* seedlings [33], in shoots of *Juniperus communis* [34], and in *Deschampsia cespitosa* plants [35]. Additionally, other studies have revealed that Cd modulates the accumulation of endogenous cytokinins in the root of *Arabidopsis thaliana*, leading to the inhibition of both root and shoot growth and causing a variety of genetic, biochemical, and physiological damages [36]. Therefore, there is a high probability that the exogenous use of naturally occurring cytokinins, such as *trans*-zeatin (*t*Z), can complement the functions of endogenous phytohormones and restore hormonal homeostasis disturbed by the toxic metal. Recent studies have confirmed this hypothesis, showing that exogenous cytokinins (i.e., kinetin and benzyladenine) enhanced growth, water status, chlorophyll accumulation, antioxidant status, stomatal opening, and the functioning of the photosynthetic apparatus in *Ricinus communis* plants subjected to copper (Cu) [37]. Similarly, cytokinins, e.g., *t*Z, kinetin, benzyladenine, and thidiazuron, have enhanced biomass production and increased chlorophyll content, which positively correlated with a higher Cd uptake efficiency in *S. alfredii* [38]. Studies performed on the green alga *A. obliquus* revealed that cytokinins increased algal tolerance to Pb stress, stimulating growth, photosynthetic pigment contents, antioxidant activity, and endogenous phytohormone levels [13,39]. Thus, the application of *t*Z to culture media may also ameliorate the negative effects of Cd on algal growth.

Increasingly, studies have indicated the presence of cytokinins in unicellular green algae and their significant role in regulating the growth and development of this group of organisms, as well as their adaptation to abiotic stress conditions [13,14,40,41,42]. However, there are no scientific reports on the participation of this group of plant hormones in the adaptation of *Desmodesmus armatus* (Chlorophyceae) to growth under abiotic stress. Despite the potential for cytokinins to improve the remediation of aquatic environments via *D. armatus*, no such roles have been elucidated for this highly adaptive species, which is commonly present in freshwater ecosystems.

Exogenous administration of cytokinin *t*Z may influence metal biosorption, endogenous levels of phytohormones, the synthesis of thiol compounds involved in metal chelation, photosynthetic pigment content, and the antioxidant machinery, thereby enhancing biochemical strategies for protecting the green alga *D. armatus* from Cd toxicity. Understanding how cytokinin induces cellular responses in aquatic microalgae to heavy metal stress has a great potential for developing successful phycoremediation techniques. Therefore, the aim of this study was to: (1) examine the optimum *t*Z concentration for algal growth and biochemical parameters, (2) investigate Cd uptake and toxicity in algal cells, and (3) assess the protective role of *t*Z against Cd stress, including algal growth, heavy metal accumulation, photosynthetic pigments, antioxidant response, PC synthesis, and hormonal homeostasis.

## 2. Materials and Methods

### 2.1. Algal Growth Conditions

*Desmodesmus armatus* (Chodat) Hegewald (Chlorophyceae) was obtained from the SAG Culture of Algae Collection (Germany; SAG Strain Number 276-4d). Homogeneous populations of young algal cells were used for subsequent experiments for growth, photosynthetic pigment, oxidative stress, antioxidant response, phytochelatin, and plant hormone determination after centrifugation at 5000× *g* for 10 min. Regular alternation of light and dark periods was adopted for complete synchronization of algal cultures [40]. The pre-cultures were grown for 7 days. After this period, algal cells reached the exponential growth phase. The cultivation of cultures for experiments was initiated by introducing algal biomass containing about 1.7 × 10^6^ (approximately 20 mL) algal cells. The initial cell density (0 h) was 5.4 × 10^6^ cells mL^−1^.

Erlenmeyer flasks containing 100 mL of autoclaved pure mineral Bold Basal Medium (BBM, pH 7.0) were applied for cultivation of *D. armatus* cultures in all tested variants [43]. BBM medium, conical flasks, and bacteriological plugs were sterilized in a heat chamber at 105 °C for 5 h. The axenic cultures were cultivated for 96 h in stable temperature (25 ± 0.5 °C) conditions. A 16:8 h light:dark period with a light intensity of 50 µmol m^−2^s^−1^ at the surface of the flasks was used as the most optimal for algal growth. The cell suspension was aerated with atmospheric air (at 1 L min^−1^) using air pumps, ensuring that the algal cultures were continuously mixed.

### 2.2. Number of Cells Determination

A Bürker chamber was used for algal growth measurements, expressed as cell number in cultures treated with *t*Z, Cd, and *t*Z + Cd, as well as in the control [13,39]. Number of algal cells was determined in each culture at 0, 24, 48, 72, and 96 h of cultivation.

### 2.3. Determination of Optimum Cytokinin Concentrations for Algal Growth and Metal Toxicity

For the purpose of analysis of the optimum concentrations of *t*Z for the growth of *D. armatus*, expressed as cell number, cytokinin was applied at the following concentrations: 0.001, 0.01, 0.1, 1, 10, and 100 µM (Figure S1). To reach these chosen concentrations, an appropriate amount of phytohormone was dissolved in 0.1 M NaOH and then added in the correct concentrations to glass flasks containing BBM medium (100 mL). Similarly, an equal volume of 0.1 M NaOH was added to the control cultures. The final concentration of NaOH in the culture media was lower than 1% *v/v*, and this amount did not exert a negative or positive effect on the growth of *D. armatus*. Each treatment consisted of four replicates, and each experiment was repeated at least twice at different times.

To identify the cadmium concentration that restricts algal growth, we applied a range from 0.01 to 100 µM Cd (Figure S1). After analyzing cell counts along with pigment, protein, and sugar levels (data not shown), we determined that 10 µM of Cd effectively inhibits growth without being excessively toxic. Therefore, this concentration was selected for in-depth analysis in subsequent experiments. At the lower end, 0.01 µM of Cd exhibited negligible toxicity, affecting neither growth nor development significantly. In contrast, 100 µM of Cd proved to be almost invariably fatal, underscoring its severe toxic potential.

All analyzed parameters were determined in each culture at 24, 48, 72, and 96 h of cultivation (except for the phytohormones level at 72 h).

### 2.4. Heavy Metal Determination

To measure the cadmium level in algal cells, the cultures were centrifuged at 9000× *g* for 10 min. Algal sediment was resuspended in 100 µM of Na_2_EDTA solution (10 mL) for 10 min for removal of Cd ions from the algal cell external surfaces. The algal suspension was then mineralized by heating at 200 °C for 40 min in a mixture of 70% nitric acid, 30% hydrogen peroxide (H_2_O_2_), and deionized water, using a Mars microwave oven (CEM Corporation, Matthews, NC, USA). A portion of this solution was evaporated to dryness at 70 °C in a quartz crucible. The dried residue was redissolved in deionized water, and the cadmium (Cd) content was measured using an electrothermal atomic absorption spectrometer, the Thermo iCE 3400, equipped with Zeeman background correction (Thermo Electron Manufacturing Ltd., Cambridge, UK) at a wavelength of 228.8 nm.

### 2.5. Photosynthetic Pigment Determination

The method provided by Zapata, et al. [44] was adopted for the qualitative and quantitative determination of photosynthetic pigments, i.e., chlorophylls, chlorophyllides, carotenes, and xanthophylls. Algal cultures (50 mL) were collected on glass filters and treated with 99.9% methanol for pigment extractions. Obtained extracts were clarified using 0.45 μm PTFE syringe cartridge filters fitted with glass prefilters (Scientific Resources Inc., Eatontown, NJ, USA). The High-Performance Liquid Chromatography (HPLC) system from Agilent 1260 Infinity Series (Agilent Technologies Inc., Santa Clara, CA, USA) was used for photosynthetic pigment determination. HPLC consisted of an autoinjector with refrigerated autosampler and contained a 500 μL sample loop for high-volume injections (300 µL), an oven for thermostatting of column Eclipse XDB C_8_ (150 × 4.6 mm; Agilent Technologies Inc., Santa Clara, CA, USA) at 25 °C, a quaternary pump with an in-line vacuum degasser, and a photo-diode array detector (DAD). The visible absorbance spectra of plant pigments were monitored using DAD at the following wavelengths: 450 nm for carotenoids and chlorophyllides, and 665 for chlorophylls with 20 nm bandwidths. The injection volume for photosynthetic pigment extract was 300 µL. Mobile phase A contained a mixture of methanol, acetonitrile, and 0.25 M aqueous pyridine solution (pH 5.0) in a proportion of 50/25/25 (*v/v/v*). Mobile phase B consisted of a mixture of methanol, acetonitrile, and acetone in a proportion of 20/60/20 (*v/v/v*). The gradient was linear, starting from 100% of eluent A and returning to 100% at the 40th minute of the experiment. Flow of the mobile phase was 1 mL min^−1^ and did not exceed a backpressure of 180 bar. The analytical data were integrated using Agilent OpenLAB ChemStation Edition C.01.09 for LC systems (Agilent Technologies Inc., Santa Clara, CA, USA).

### 2.6. Malondialdehyde and Hydrogen Peroxide Content Determination

Lipid peroxidation was determined by measuring the total malondialdehyde (MDA) content, a marker of the lipid peroxidation process in algal cells, as previously described by Heath and Packer [45] and detailed by Piotrowska-Niczyporuk, et al. [39]. The level of hydrogen peroxide (H_2_O_2_) in *D. armatus* cells (10 mL) was measured using the method outlined by Alexieva, et al. [46]. For MDA and H_2_O_2_ determination, algal cells were disintegrated in 0.1% (*w*/*v*) trichloroacetic acid (TCA) and homogenized in a bead mill (50 Hz, 10 min, TissueLyser LT; Qiagen GmbH, Düsseldorf, Germany). Algal extracts were used for spectrophotometrical (Hitachi U-5100 UV-Vis spectrophotometer; Hitachi High-Tech Science Corporation, Tokyo, Japan) measurement of oxidative marker levels [34,40].

### 2.7. Determination of Ascorbate and Proline

The total ascorbate content in *D. armatus* cells was determined using the method described by Kampfenkel, et al. [47]. Cells were disrupted using 0.1 M phosphate buffer solution (pH = 2.0) in a bead mill (50 Hz, 10 min, TissueLyser LT; Qiagen GmbH, Düsseldorf, Germany). The extraction procedure of proline (Pro) started with homogenization of algal cells in 100 mM of phosphate buffer (pH 7.5) in a bead mill. The level of Pro in algal extracts was determined spectrophotometrically (Hitachi U-5100 UV-Vis spectrophotometer; Hitachi High-Tech Science Corporation, Tokyo, Japan) at a wavelength of 546 nm after reaction with an acidic 2.5% ninhydrin [48]. The proline concentration was calculated based on the cell number.

### 2.8. Determination of the Antioxidant Enzymes’ Activities

The same extraction solution (0.05 M phosphate buffer, pH 7.8) was used for analysis of the antioxidant enzyme activity. Algal cells (50 mL) were centrifuged at 5000× *g* for 10 min. Cell suspension was homogenized in a bead mill (50 Hz, 10 min, TissueLyser LT; Qiagen GmbH, Düsseldorf, Germany) in 1 mL of 0.05 M phosphate buffer. After homogenization, the suspension was centrifuged at 12,000× *g* for 10 min at 4 °C. The obtained supernatant was used for enzyme assays. The activity of superoxide dismutase (SOD; EC 1.15.1.1) was determined spectrophotometrically (Hitachi U-5100 UV-Vis spectrophotometer; Hitachi High-Tech Science Corporation, Tokyo, Japan) by measuring the inhibition of the photochemical reduction of substrate nitroblue tetrazolium (NBT) at a wavelength of 560 nm [49]. One unit (U) of SOD activity, calculated per mg of protein, is defined as the amount that causes 50% inhibition of NBT photochemical reduction. The activity of catalase (CAT, EC 1.11.1.6) was measured by monitoring the absorbance decrease at 240 nm because of the H_2_O_2_ disappearance (ε = 39.4 mM^−1^ cm^−1^) at 240 nm [50]. One unit (U) of CAT activity is considered the amount of enzyme that decomposes 1 µmol of H_2_O_2_ per mg of soluble protein per minute at the stable temperature conditions (30 °C). The activity of ascorbate peroxidase (APX; EC 1.11.1.1) was determined following the method by Nakano and Asada [51] by the observation of the changes in absorbance at 290 nm (ɛ = 2.8 mM^−1^ cm^−1^). One unit (U) of APX was calculated as the amount of enzyme that oxidizes 1 µmol of substrate ascorbic acid that is consumed per minute per mg of soluble protein (30 °C). Glutathione reductase (GR; EC 1.8.1.7) activity was measured based on the rate of nicotinamide adenine dinucleotide phosphate oxidation observed at 340 nm. The enzyme activity (U) was expressed as ∆ A340 per mg of soluble protein per minute at the stable temperature conditions (25 °C) [52]. All spectrophotometric measurements of the activity of antioxidant enzymes were performed using a thermostatted Hitachi U-5100 UV-Vis Spectrophotometer (Hitachi High-Tech Science Corporation, Tokyo, Japan).

### 2.9. Determination of Cysteine, γ-Glutamylcysteine, Glutathione, and Phytochelatins

To determine the content of PCs and their precursors (cysteine, Cys; *γ*-glutamylcysteine, *γ*-Glu-Cys; glutathione, GSH), 50 mL of algal cells were harvested by centrifugation for 10 min at 5000× *g*. The suspension of algal cells was resuspended in 1 mL of 0.1% trifluoroacetic acid (TFA; *w*/*v*) with the addition of 6.3 mM of diethylenetriaminepentaacetic acid (DTPA). Then, *D. armatus* cultures were homogenized in a bead mill (50 Hz, 10 min, TissueLyser LT; Qiagen GmbH, Düsseldorf, Germany) for cell disruption. After centrifugation (10 min, 9000× *g*, and 4 °C), the obtained supernatant was kept on ice before derivatization reaction and thiol analysis [53]. Thiol compounds were derivatized with monobromobimane (mBBr) as a fluorescent tag. The samples were stored in the dark at 4 °C until qualitative and quantitative analysis using the HPLC system (Agilent Technologies Inc., Santa Clara, CA, USA). Thiol compounds, such as Cys, *γ*-Glu-Cys, GSH, and PCs, were separated on a COSMOSIL Packed Column 5C-18-MS-II (4.6 μm × 250 mm; Nacalai Inc., San Diego, CA, USA) at a temperature of 37 °C in a column compartment (oven). The sample was injected (25 µL) using a thermostatted autosampler (4 °C) and thiol groups were eluted using a slightly concave gradient from 12% to 25% (*v*/*v*) of methanol with 1% TFA over 15 min, followed by a linear gradient from 25% to 35% methanol with 1% TFA from 15 to 29 min, and then from 35% to 50% methanol with 1% TFA from 29 to 60 min to post-injection. Fluorescence of derivatized groups was monitored by an Agilent 1260 Infinity Fluorescence Light Detector (Agilent Technologies Inc., Santa Clara, CA, USA) at an excitation wavelength of 380 nm and emission wavelength of 470 nm. The retention times of PC precursors (Cys, *γ*-Glu-Cys, and GSH) and PC oligomers were confirmed using their standards (AnaSpec, Fremont, CA, USA). The obtained chromatograms were analyzed using Agilent OpenLAB ChemStation Edition C.01.09 for LC systems (Agilent Technologies Inc., Santa Clara, CA, USA).

### 2.10. Phytochelatin Synthase Activity Assay

The activity of phytochelatin synthase (PCS) was determined in algal cells following a modified protocol by Finkemeier, et al. [54]. Algal cell suspension (50 mL) was centrifuged for 10 min at 5000× *g*. Then, cells were disintegrated in 2 mL of buffer containing 20 mM of HEPES-NaOH (pH 7.5), 10 mM of *β*-mercaptoethanol, 20% glycerol, and 100 mg of polyvinylpyrrolidone in a bead mill homogenizer (50 Hz, 10 min, TissueLyser LT; Qiagen GmbH, Düsseldorf, Germany). After centrifugation at 13,000× *g* for 10 min at 4 °C, the obtained supernatant was used for PCS activity measurements. The assay mixture included 400 µL of the extract, 100 µL of reaction buffer (100 µM of CdSO_4_, 25 mM of GSH, 10% glycerol, and 250 mM of HEPES-NaOH, pH 8.0), and a protease inhibitor mix ‘Complete’ (Sigma-Aldrich, St. Louis, MO, USA). Then, thiol groups were derivatized using mBBr, and HPLC analysis was performed as described earlier.

### 2.11. Determination of Plant Hormone Content

Phytohormone standards, including abscisic acid (ABA), indole-3-acetic acid (IAA), indole-3-butyric acid (IBA), phenylacetic acid (PAA), brassinolide (BL), epibrassinolide (epiBL), castasterone (CS), 24-epicastasterone (epiCS), cathasterone (CT), 28-homobrassinolide (HBL), typhasterol (TY), 6-deoxytyphasterol (6dTY), *cis*-zeatin (*c*Z), and others, were acquired from OlChemIm (Olomouc, Czech Republic). For phytohormone level measurement, algal cells were centrifuged (10 min at 5000× *g*). Then, cell suspension was resuspended in 1 mL (*v/v*) of 50% acetonitrile (ACN) and homogenized in a bead mill (50 Hz, 10 min, TissueLyser LT; Qiagen GmbH, Düsseldorf, Germany). Subsequently, samples underwent further homogenization using an ultrasound processor VCX 130 (maximum power 130 W, maximum frequency 20 kHz, 5 min; Sonics & Materials, Inc., Newtown, CT, USA). The extracts were mixed in a laboratory shaker (90 rpm, dark, 5 °C, 30 min; LC-350, Pol-Eko-Aparatura, Gliwice, Poland). The obtained extracts from algal cells were then processed using Waters SPE Oasis^®^ HLB cartridges, which were activated and equilibrated with 1 mL of 100% methanol, 1 mL of H_2_O, and 1 mL of 50% can [55]. Samples were subsequently evaporated to dryness in a centrifugal vacuum concentrator (Labconco CentriVap Micro IR, Kansas City, MO, USA), redissolved in 50 μL of 30% ACN, and transferred into the insert vials.

The quantitative and qualitative analysis of phytohormones was performed using the LCMS-8050 system (Shimadzu Corporation, Kyoto, Japan), comprising an autosampler, binary pump, degasser, thermostatted column compartment, and triple-quadrupole mass spectrometer. A 10 μL sample volume was injected onto a Waters XSelect C_18_ column (250 mm × 3.0 mm, 5 μm), maintained at 50 °C in a column oven. The mobile phases consisted of 0.01% formic acid (FA) in ACN (mobile phase A), and 0.01% FA in H_2_O (mobile phase B). The flow rate of mobile phase was 0.5 mL min^−1^. Separation occurred in ESI-positive mode with a gradient from 5% to 100% A over 28 min. LabSolution software 5.6 version (Shimadzu Browser Workstation Software for LC-MS) controlled the instrument and managed MS data acquisition and processing. The content of phytohormones in algal cells was measured at the 72 h mark, coinciding with the peak of algal growth influenced by *t*Z alone and in combination with Cd.

### 2.12. Statistical Analysis

Each measurement was performed with four replicates, and each experiment was conducted at least twice at different times. Mean comparisons were conducted using Tukey’s post-hoc test (TIBCO Software Statistica version 13.3). The level of significance was set at *p* < 0.05 for all statistical tests and comparisons.

## 3. Results and Discussion

Microalga *D. armatus*, belonging to the Chlorophyceae class, plays a key role as a primary producer and is commonly found in most freshwater ecosystems. The use of green algae for remediation of waters polluted by heavy metals, such as Cd, has garnered significant attention in recent years [3,5,10]. Heavy metals are known for their high toxicity to microalgae, potentially reducing their growth, development, and viability. Additionally, the presence of toxic metals can alter the phytoplankton community and impact the functioning of aquatic environments. One possible reason for the toxic effects of heavy metals on algal growth could be the depletion of endogenous cytokinins, which are crucial in regulating and stimulating cell proliferation, as reported in previous experiments [13,40]. To address this issue, the feasibility of adding exogenous cytokinin, specifically *trans*-zeatin (*t*Z), to enhance microalgal growth and adaptation strategies to abiotic stress generated by Cd ions, was evaluated.

### 3.1. Algal Growth

**Figure 1:**
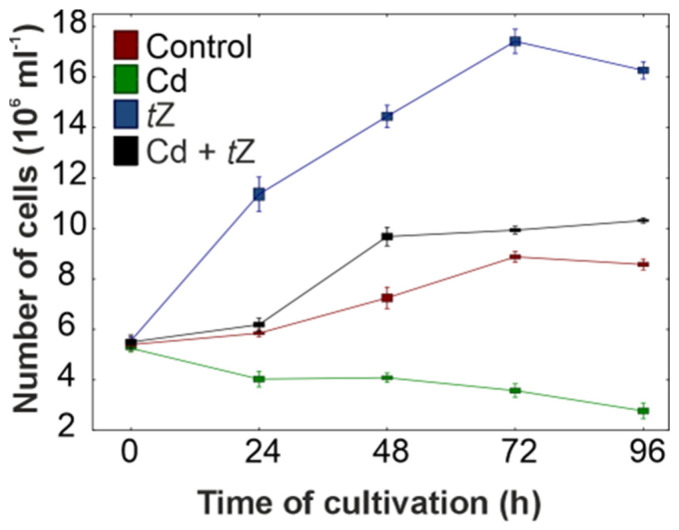
The effect of cadmium (Cd) and *trans*-zeatin (*t*Z) on cell number in relation to the control at 0, 24, 48, 72, and 92 h of culture. Data are the means of four independent experiments ± SD.

Algal growth, a commonly used parameter in the ecotoxicological risk assessment of heavy metals in aquatic environments, has been extensively studied. Results show that increasing concentrations of heavy metals in the medium can accumulate in algal cells and negatively affect their growth [1,2,3,5,6,8]. *D. armatus* cultures responded negatively to increasing concentrations of Cd from 0.01 µM to 100 µM, as assessed by cell counts over 96 h of experimentation. For instance, a growth inhibition of 67.7%, expressed as cell number, was observed in cultures exposed to 10 µM of Cd at the 96 h mark of cultivation (Figure 1, Figure S1).

A concentration of 100 µM of Cd proved extremely toxic, resulting in an 89.9% reduction in cell number. Consequently, Cd at 10 µM was chosen for subsequent experiments. The ability of heavy metals to inhibit microalgal growth depends on the metal concentration, the specific species of algae, and the exposure time [6,7,56]. Growth reduction in response to Cd has been reported for many algal species, such as *Chlorella vulgaris* [56], *Chlamydomonas reinhardtii* [12,57], *A. obliquus*, *Chlorella pyrenoidos*a, and *Selenastrum capricornutum* [58]. The higher inhibition of *D. armatus* growth observed in our studies with increased Cd concentrations may be explained by its damaging effects on algal cell morphology and physiology [40]. Moreover, high concentrations of Cd inhibit the synthesis of chlorophylls, damage the photosynthetic apparatus, and trigger oxidative stress, leading to a decrease in the algal growth rate.

Cytokinins are potent phytohormones that stimulate cell division in plants and algae. Therefore, the effect of *t*Z at a range of concentrations (0.001–100 µM) on the cell number of *D. armatus* was investigated over 96 h of cultivation (Figure 1). This part of the experiment aimed to select the optimal cytokinin concentration for algal growth under stress conditions. The highest biological activity of *t*Z occurred at a concentration of 1 µM, as cell numbers increased by 99.2% and 96.2% at 48 and 72 h of the experiment, respectively, compared to the control. Thus, *t*Z at 1 µM was chosen for subsequent experiments. Our results confirmed that cytokinin *t*Z can act as signaling molecules during cell-cycle progression in *D. armatus* cultures, aligning with previous observations where exogenous cytokinins stimulated cell proliferation in green algae, such as *A. obliquus* [39] and *Chlorella variabilis* [59].

In the next step, the effect of 1 µM of *t*Z in combination with 10 µM of Cd on cell number in *D. armatus* cultures was examined (Figure 1). The results indicated that exogenous *t*Z alleviated the toxic effects of 10 µM of Cd and stimulated algal growth. For example, 1 µM of *t*Z in combination with the heavy metal increased cell numbers by 5.7%, 33.5%, 11.9%, and 20.3% at 24, 48, 72, and 96 h of cultivation, respectively, compared with the control. Although the exogenous application of *t*Z prevented growth inhibition and even stimulated cell numbers above the control, the mechanisms behind this process are yet to be fully understood. This positive effect of cytokinin on cell number growing in the presence of Cd could serve as a protective mechanism against abiotic stress and improve industrial water remediation efforts. The positive effect of cytokinins on growth promotion in the presence of Cd has been well documented for vascular plants, e.g., *S. alfredii* [31,38], *Triticum aestivum* [60], *Vigna angularis* [61], and the alga *Euglena gracilis* [27].

### 3.2. Cd Uptake

The accumulation of Cd ions from the nutrient medium by *D. armatus* cells was time-dependent, with levels increasing from 76.25 to 121.03 amol cell^−1^ over 96 h of cultivation (Table 1). Furthermore, the uptake of Cd ions was stimulated by the exogenous application of *t*Z at a concentration of 1 µM, rising from 83.61 to 145.16 amol cell^−1^ during the 96 h experiment. This resulted in a higher metal removal yield, which is the primary objective of phycoremediation in freshwater ecosystems. Our results indicated that *t*Z plays a crucial role in enhancing both the growth of *D. armatus* and its efficiency in Cd uptake. However, the mechanisms underlying Cd biosorption by *D. armatus* and its stimulation by exogenous cytokinins remain unclear. It is speculated that metal transport through cell membranes and its cellular translocation may be enhanced in the presence of exogenous *t*Z.

Similar to our findings, Cd accumulation in the vascular plant *S. alfredii* was significantly higher under the influence of cytokinins. Cytokinin *t*Z at a concentration of 10 µM was most effective in enhancing biomass production, which correlated with a higher Cd uptake efficiency in *S. alfredii* [31,38]. Conversely, a reduced Cd uptake was observed with the application of another cytokinin (kinetin) to *V. angularis* plants. Exogenous cytokinin mitigated the harmful effects of Cd stress by promoting plant growth and enhancing defense mechanisms against abiotic stress [61]. Therefore, the effect of cytokinin on metal uptake strongly depends on the specific plant species and the phytohormone used. In summary, *t*Z can serve as an effective signaling molecule to enhance algal growth and cadmium bioremediation efficiency in the green alga *D. armatus*.

### 3.3. Photosynthetic Pigments

**Figure 2:**
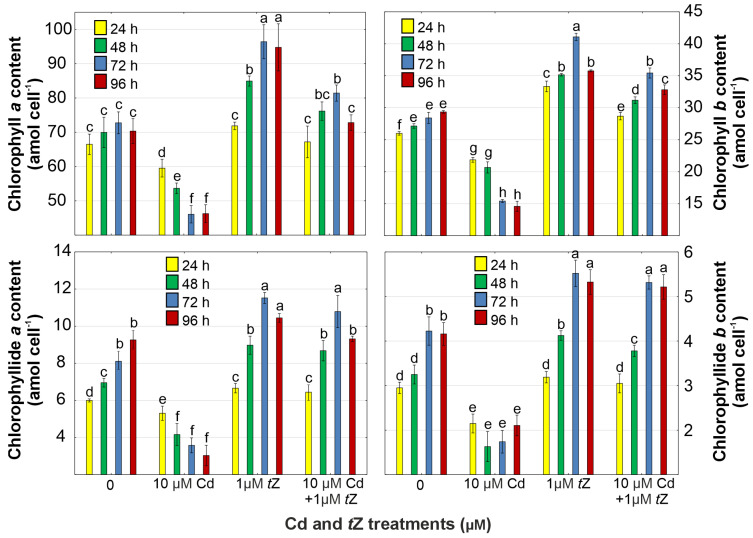
The effect of cadmium (Cd) and *trans*-zeatin (*t*Z) on chlorophyll *a* and *b*, as well as chlorophyllide *a* and *b* content in relation to the control at 24, 48, 72, and 92 h of culture. Data are the means of four independent experiments ± SD. Treatments with at least one letter the same were not significantly different according to Tukey’s post-hoc test.

The analysis of photosynthetic pigments (chlorophylls, chlorophyllides, and carotenoids), crucial for the photosynthesis process in algal cells, is commonly utilized as an endpoint in assessing the toxicity of environmental pollutants, such as Cd. The results of *D. armatus* indicated that chlorophylls *a* and *b* are particularly sensitive to the presence of heavy metals in growth media (Figure 2, Figures S2–S5). Chlorophyll *a* content in *D. armatus* cells was reduced by 10.5%, 23.3%, 36.7%, and 34.2% in response to Cd exposure over 24, 48, 72, and 96 h of cultivation, respectively. Similarly, chlorophyll *b* levels decreased by 15.92%, 23.8%, 45.7%, and 50.3% under the same conditions. These findings suggest that Cd may inhibit chlorophyll synthesis due to the reduced content of chlorophyllides, essential in the synthesis of these photosynthetic pigments (Figure 2, Figures S2–S5).

The detrimental effect of Cd on chlorophyll accumulation has been corroborated in algal species, such as *C. pyrenoidosa* and *Scenedesmus acutus* [11], as well as in *C. reinhardtii* [12]. The Cd-induced reduction in chlorophyll levels in algal cells might also be related to the metal’s impact on oxidative stress or peroxidation of thylakoid lipids, leading to chlorophyll degradation. Conversely, the exogenous application of *t*Z can mitigate metal toxicity on the photosynthetic apparatus in *D. armatus*. The addition of *t*Z to growth media enhanced the contents of chlorophylls and their bio-precursors in microalgae exposed to Cd stress (Figure 2). Notably, increases by 11.9% in chlorophyll *a* content, 44.5% in chlorophyll *b* levels, 32.0% in chlorophyllide *a* levels, and 25.8% in chlorophyllide *b* amount were observed at 72 h of cultivation in algal cells exposed to Cd in combination with *t*Z.

The protective effect of cytokinins during metal stress aligns with findings in vascular plants. For instance, the exogenous application of kinetin significantly ameliorated the damaging effects of Cd on chlorophylls in *Solanum lycopersicum* [62], *V. angularis* [61], and *S. alfredii* [31,38]. Additionally, benzyladenine mitigated the toxic impact of copper (Cu) on the photosynthetic apparatus in *R. communis* [37]. Similar improvements in chlorophyll levels were observed in green algae, such as *C. vulgaris* exposed to Cd, Cu, or Pb [63] and *A. obliquus* treated with Pb [39], likely due to cytokinin-induced activation of the antioxidant system protecting the photosynthetic apparatus from Cd-induced oxidative damage.

Carotenoids, divided into carotenes and xanthophylls, are vital pigments involved in photosynthesis, alongside chlorophylls, also serving as photo-protectors, antioxidants, and precursors of plant hormones [64]. Increases in carotene contents over the control were observed in all tested variants, with the highest accumulation in cultures treated with both cytokinin *t*Z and Cd. Co-application of cytokinin with the heavy metal increased *α*-carotene levels by 73.4%, whereas *β*-carotene was stimulated by 54.6% at 72 h of cultivation (Table 2, Figures S2–S5). It can be speculated that the increase in levels of *α*- and *β*-carotene, known for their antioxidant properties, in response to *t*Z combined with Cd, is part of a strategy activated in *D. armatus* cells to counteract the toxic effect of this heavy metal on the photosynthetic apparatus. The protective effects of phytohormones on photosynthesis were also observed in the green alga *A. obliquus* exposed to Pb with *t*Z, kinetin, and diphenylurea [39], as well as in *V. angularis* treated with kinetin and Cd [61]. Thus, carotenes are crucial antioxidants in minimizing abiotic stress generated by Cd in algal cells.

A similar trend was observed with other carotenoids–xanthophylls (violaxanthin, antheraxanthin, and zeaxanthin), whose levels were stimulated in *D. armatus* cells growing in the presence of *t*Z and Cd alone, and when *t*Z was applied with Cd (Table 2, Figures S2–S5). The highest accumulations of antheraxanthin, violaxanthin, and zeaxanthin, respectively, during 96 h of cultivation, were observed in response to a mixture of 1 µM of *t*Z with 10 µM of Cd, compared to the control.

Pigments belonging to the xanthophyll cycle are known for their high efficiency in free radical quenching during photosynthesis and photo-inhibitory properties and, thereby, may protect the photosynthetic apparatus against oxidative stress induced by various chemical toxicants [12,40,61]. The positive effect of cytokinins on the activation of the xanthophyll cycle was confirmed in other algal species, such as *A. obliquus* growing in the presence of Pb [39]. The protective effect of different cytokinins on the photosynthetic machinery was also confirmed in *S. alfredii* hyperaccumulating plants growing in the presence of Cd [31,38]. Therefore, the enhanced accumulation of xanthophylls observed in *D. armatus* exposed to exogenous cytokinin might be part of the algal acclimatization strategy against oxidative stress generated by Cd ions.

### 3.4. Oxidative Stress

**Figure 3:**
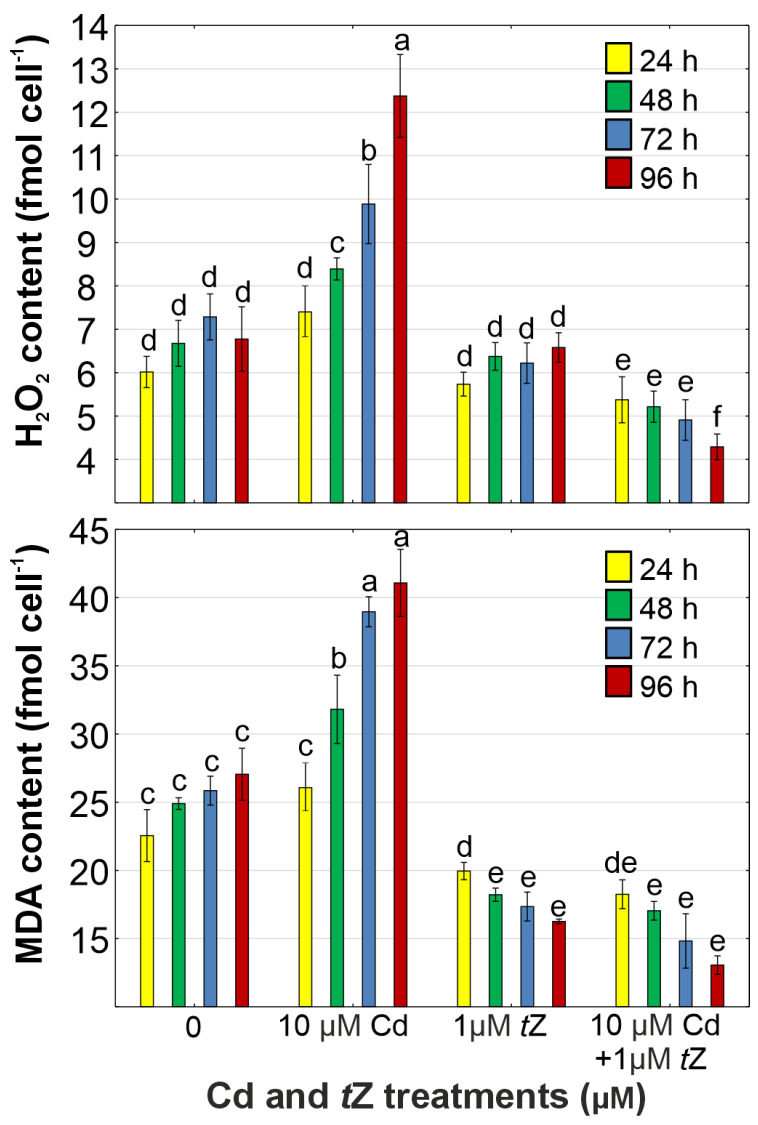
The effect of cadmium (Cd) and *trans*-zeatin (*t*Z) on hydrogen peroxide (H_2_O_2_) and malondialdehyde (MDA) content in relation to the control at 24, 48, 72, and 92 h of culture. Data are the means of four independent experiments ± SD. Treatments with at least one letter the same were not significantly different according to Tukey’s post-hoc test.

Compared to unstressed *D. armatus* cells, significant increases in the contents of H_2_O_2_ (by 82.6%) and MDA (by 51.9%) were observed in cultures exposed to 10 µM of Cd at 96 h of cultivation (Figure 3). Oxidative stress induced by heavy metals has been reported in many vascular plant species [12,57] and algae, such as *C. vulgaris* [3], *A. obliquus*, *C. pyrenoidosa*, and *S. capricornutum* [58]. Consequently, Cd disrupts the redox homeostasis, thereby affecting the cell functioning and tolerance potential of alga *D. armatus*.

The application of *t*Z significantly reduced the accumulation of ROS, including H_2_O_2_ (by 36.7%), and inhibited lipid peroxidation (by 51.7%), as expressed by the MDA content in *D. armatus* cells growing under Cd stress at 96 h of cultivation compared with the control (Figure 3). Therefore, the protective effect of *t*Z against oxidative stress in algal cells generated by the heavy metal was confirmed.

The beneficial role of these phytohormones in minimizing symptoms of oxidative stress caused by the presence of heavy metals in the environment has been well documented for vascular plants [60,61]. For instance, the exogenous application of benzyladenine decreased the contents of H_2_O_2_ and MDA, as well as electrolyte leakage in *Solanum melongena* exposed to Zn stress [18]. Similarly, the application of kinetin reduced H_2_O_2_ synthesis in *S. lycopersicum* treated with Cd [62]. In line with our findings, previous studies have also shown that the exogenous application of cytokinins inhibited ROS formation in other green algae, such as *C. vulgaris* exposed to Cd, Cu, or Pb [63] and *A. obliquus* treated with Pb [39]. Our results indicate that this cytokinin acts as an efficient signaling molecule accelerating the biochemical mechanisms involved in ROS scavenging in algal cells under abiotic stress.

### 3.5. Antioxidant Response

**Figure 4:**
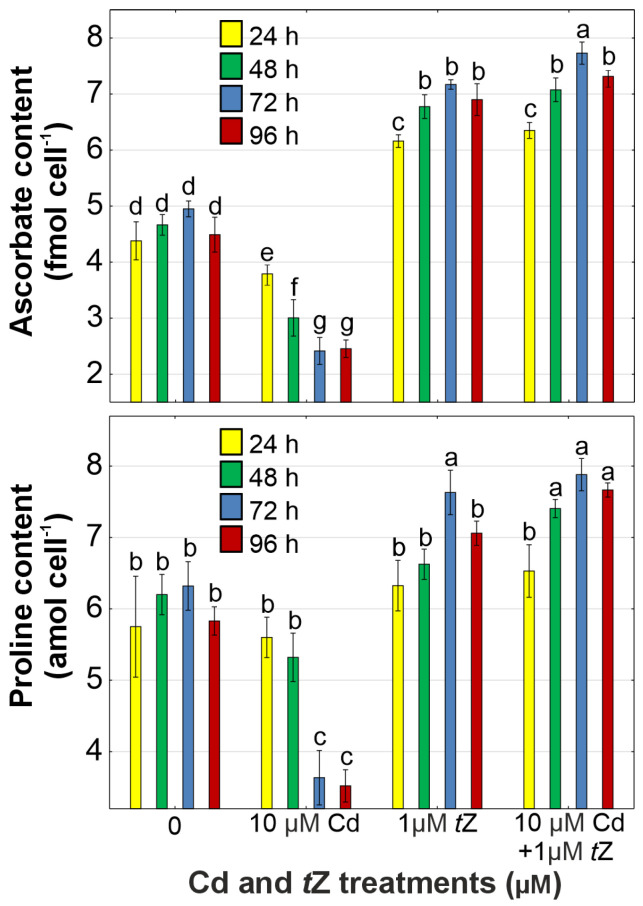
The effect of cadmium (Cd) and *trans*-zeatin (*t*Z) on ascorbate and proline content in relation to the control at 24, 48, 72, and 92 h of culture. Data are the means of four independent experiments ± SD. Treatments with at least one letter the same were not significantly different according to Tukey’s post-hoc test.

The cytokinin-mediated reduction in Cd-induced oxidative stress in the green alga *D. armatus* may be attributed to an enhanced antioxidant defense system. Ascorbate and proline (Pro) are vital non-enzymatic antioxidants in algae and vascular plants. Our results revealed that Cd decreased the content of total ascorbate and Pro by 51.2% and 42.5%, respectively, at 72 h of cultivation, compared to the control (Figure 4). A significant increase in ascorbate and Pro levels by 62.9% and 31.5%, respectively, was observed after co-application of *t*Z with Cd to *D. armatus* cultures at 96 h of the experiment. Higher contents of antioxidants in algal cells treated with *t*Z were well correlated with the inhibition of H_2_O_2_ production and lipid peroxidation processes, as well as with enhanced algal adaptation to the presence of heavy metals in the aquatic environment.

The positive effect of cytokinins on the synthesis of ascorbate and Pro has also been confirmed in green algae, such as *C. vulgaris* [63] and *A. obliquus* [39], growing in the presence of heavy metals. Similarly, benzyladenine stimulated the contents of antioxidants in *S. melongena* treated with Zn [18], and *t*Z enhanced the non-enzymatic antioxidants’ level in Cd-treated *T. aestivum* [60]. These observations indicate that cytokinins may enhance the antioxidant system against metal-induced ROS burst in both algal cells and vascular plants, leading to higher tolerance to abiotic stress conditions.

**Figure 5:**
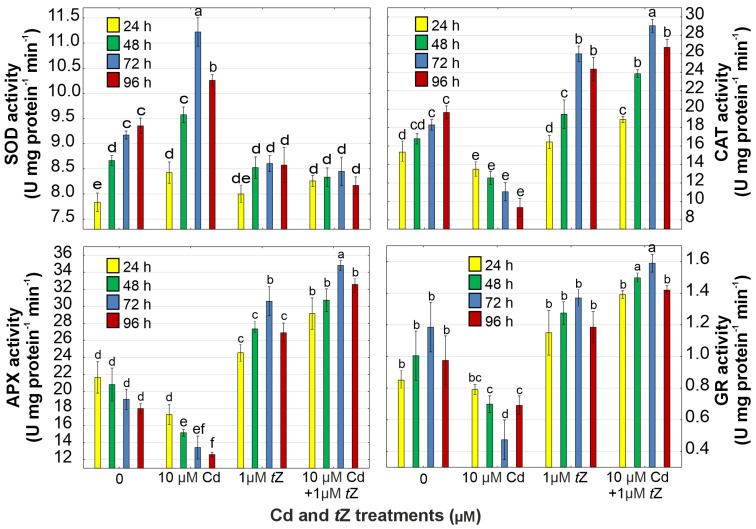
The effect of cadmium (Cd) and *trans*-zeatin (*t*Z) on superoxide dismutase (SOD), catalase (CAT), ascorbate peroxidase (APX), and glutathione reductase (GR) activity in relation to the control at 24, 48, 72, and 92 h of culture. Data are the means of four independent experiments ± SD. Treatments with at least one letter the same were not significantly different according to Tukey’s post-hoc test.

SOD, CAT, APX, and GR are major antioxidant enzymes involved in ROS scavenging and the regulation of cellular redox status (Figure 5). The activity of SOD was stimulated by 22.3% in *D. armatus* exposed to 10 µM of Cd, while the activities of CAT, APX, and GR were inhibited by 39.6%, 29.7%, and 60.0%, respectively, at 72 h of cultivation, compared to the control. The increase in SOD activity may be related to H_2_O_2_ production since the enzyme catalyzes the dismutation reaction of the superoxide radical into H_2_O_2_. Therefore, the disruption in antioxidant enzyme activity in algal cells treated with Cd led to ROS generation and enhanced lipid peroxidation. The application of 1 µM of *t*Z + 10 µM of Cd increased SOD, CAT, APX, and GR activities by 7.9%, 58.7%, 82.3%, and 34.1%, respectively, at 72 h of cultivation compared to the control.

SOD plays a ubiquitous role in neutralizing superoxide and forming H_2_O_2_, while CAT scavenges H_2_O_2_ in the peroxisomes, mitochondria, cytosol, and chloroplasts. APX and GR are crucial enzymes of the ascorbate–glutathione cycle present in chloroplasts involved in H_2_O_2_ detoxification, thus protecting the organelle during abiotic stress [15]. Higher activity of APX and GR, along with increased contents of total ascorbate and GSH in *D. armatus* cells, supports the hypothesis that exogenous cytokinin enhances the ascorbate–glutathione cycle in response to heavy metal exposure. Similarly, benzyladenine increased the levels of both ascorbate and GSH, stimulating activities of enzymes involved in the ascorbate–glutathione cycle, such as APX and GR, in *S. melongena* seedlings growing in the presence of Zn [18]. Antioxidant enzyme activities, including SOD, CAT, and components of the ascorbate–glutathione cycle, significantly increased due to kinetin treatment in *V. angularis* plants subjected to Cd [61]. Furthermore, antioxidant enzyme activities were enhanced in *T. aestivum* seedlings exposed to *t*Z and Cd, leading to higher plant tolerance, increased photosynthesis efficiency, and better productivity [60].

Different antioxidant enzymes play specific roles and are located in various parts of the algal cell. Therefore, the process of H_2_O_2_ scavenging in cells treated with cytokinin may be more efficient in a polluted aquatic environment. Treating algal cells with *t*Z enhanced the activity of antioxidant enzymes, leading to a reduction in ROS levels and the lipid peroxidation process stimulated by Cd. Thus, *t*Z alleviated the toxic effects of heavy metals on algal growth and viability through the activation of the antioxidant machinery. These observations confirm our results obtained on algae, such as *C. vulgaris* and *A. obliquus* [39,63], suggesting that cytokinins may modulate heavy metal stress in *D. armatus* cells via precise regulation of harmful ROS levels. The more efficient functioning of antioxidant enzymes in *t*Z-treated algal cultures may have contributed to greater protection of cells against Cd toxicity. In summary, *t*Z activates the antioxidant defense capacity in microalgae during oxidative stress induced by Cd, enabling *D. armatus* to survive in challenging environments.

### 3.6. Phytochelatin Synthesis

**Figure 6:**
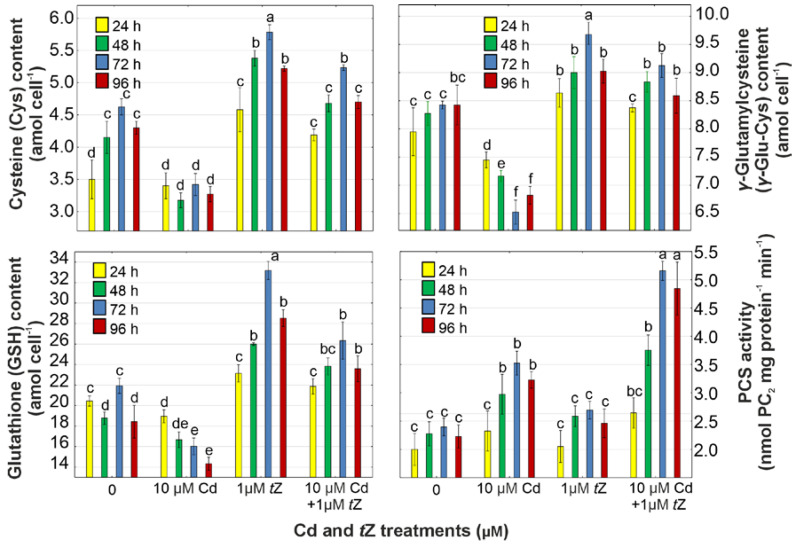
The effect of cadmium (Cd) and *trans*-zeatin (*t*Z) on the content of cysteine (Cys), *γ*-glutamylcysteine (*γ*-Glu-Cys), glutathione (GSH), and phytochelatin synthase (PCS) activity in relation to the control at 24, 48, 72, and 92 h of culture. Data are the means of four independent experiments ± SD. Treatments with at least one letter the same were not significantly different according to Tukey’s post-hoc test.

In *D. armatus* cultures exposed to Cd, the levels of PC precursors (Cys, *γ*-Glu-Cys, and GSH) were reduced by 26.1% (Cys), 22.6% (*γ*-Glu-Cys), and 26.9% (GSH) compared to the control at 72 h of cultivation (Figure 5). However, cells treated with *t*Z under Cd stress showed increases in the levels of Cys, *γ*-Glu-Cys, and GSH by 13.2%, 8.3%, and 28.0%, respectively (Figure 6, Figures S6–S9).

*γ*-Glu-Cys is synthesized from glutamate (Glu) and Cys, after which glycine (Gly) is added to the C-terminal of *γ*-Glu-Cys, leading to the synthesis of GSH, as observed in *S. acutus* exposed to chromium (Cr) ions [65]. This tripeptide serves various physiological functions as an antioxidant molecule involved in redox regulation, metabolite conjugation, and detoxification of xenobiotics. GSH is particularly important for PC synthesis, providing the *γ*-Glu-Cys moiety to the enzyme PCS [22,66]. Additionally, the increase in the GSH level in *D. armatus* cells in response to *t*Z + Cd may also be due to the higher activity of GR, responsible for the recycling pathways of GSH under stress conditions. The *t*Z-induced increase in GSH content within the Cd-treated algae suggests that cytokinin alters the synthesis and/or regeneration of this tripeptide, leading to higher algal tolerance to unfavorable environmental conditions. Our findings are in line with previous studies, showing that cytokinin treatment accelerated GSH synthesis in algae *A. obliquus*, resulting in enhanced adaptation to Pb stress [13,39], and better acclimatization of *T. aestivum* seedlings to Cd toxicity [60].

PCs are synthesized from GSH in a transpeptidation reaction catalyzed by PCS, a constitutively expressed enzyme activated by metal ions [17,19,66]. Our results show that Cd increased PCS activity from 16.3% to 47.2% during 96 h of cultivation in *D. armatus* cells compared to the control (Figure 6, Figures S6–S9). Notably, the highest enzyme activity was observed in cells exposed to *t*Z in combination with Cd, with an increase of 115.4% at 72 h of cultivation compared to the control. This suggests that exogenous cytokinin is an efficient signaling molecule capable of activating PCS, thereby leading to higher PC production in *D. armatus* cells subjected to Cd stress. The increased PCS activity in algae exposed to both *t*Z and Cd correlated with higher concentrations of all thiol compounds because the polymerization of PC_2_ into PC_3_, PC_4_, and PC_5_ was a direct result of enhanced PCS activity [13]. Higher enzyme activity correlates with higher Cd uptake induced by exogenous cytokinin. Our observed trends align with previous studies on freshwater algae, demonstrating that PCS activity increases with the intracellular heavy metal concentration [40]. Hence, higher PCS activity under the influence of exogenous *t*Z was associated with greater Cd accumulation in *D. armatus* cells. Our results concur with data indicating that another cytokinin, *trans*-zeatin riboside, increased resistance to Cd and Zn in the halophyte plant species *Kosteletzkya pentacarpos*, leading to the synthesis of GSH and PCs [67]. It can be speculated that *t*Z enhances PCS activity in *D. armatus* cultures growing in the presence of Cd, thereby reducing metal toxicity through increased PC production, resulting in higher cellular tolerance and phycoremediation properties of this algal species.

**Figure 7:**
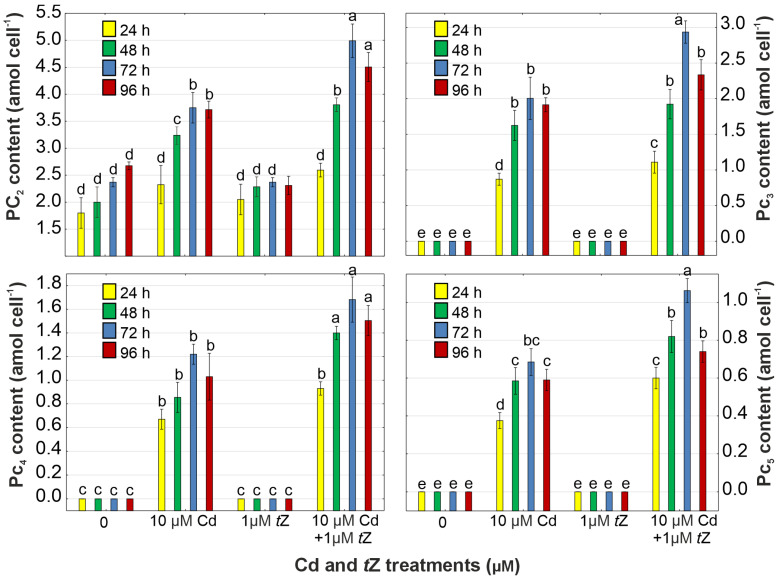
The effect of cadmium (Cd) and *trans*-zeatin (*t*Z) on the content of phytochelatin oligomers (PC_2_, PC_3_, PC_4_, and PC_5_) in relation to the control at 24, 48, 72, and 92 h of culture. Data are the means of four independent experiments ± SD. Treatments with at least one letter the same were not significantly different according to Tukey’s post-hoc test.

PCs, non-protein thiols synthesized in vascular plants and algae, play key roles as metal chelators induced by the presence of heavy metals, especially Cd [17,19,66]. Our observations support this hypothesis, as the contents of PC_2_, PC_3_, PC_4_, and PC_5_ increased to 3.75 amol cell^−1^, 2.01 amol cell^−1^, 0.87 amol cell^−1^, and 0.69 amol cell^−1^, respectively, in *D. armatus* cells exposed to Cd at 72 h of the experiment (Figure 7, Figures S6–S9).

Moreover, exogenous *t*Z effectively stimulated the production of PCs, capable of forming stable metal–PC complexes under Cd stress. Consequently, *D. armatus* cells exhibited higher accumulation of PC_2_ (4.99 amol cell^−1^), PC_3_ (2.49 amol cell^−1^), PC_4_ (1.68 amol cell^−1^), and PC_5_ (1.06 amol cell^−1^) in response to *t*Z combined with Cd.

The co-application of cytokinin with Cd led to a higher PC content, and the accumulation of these thiol peptides positively correlated with enhanced Cd uptake by the microalga. Therefore, cytokinins may be involved in the regulation of signaling pathways leading to PC synthesis under heavy metal stress, potentially reducing metal toxicity through increased PC production in algal cells. However, the role of exogenous cytokinins in plant adaptation to heavy metals is still poorly understood, with only a few studies available regarding the relationship between cytokinin and PC synthesis. The link between cytokinins and PC accumulation under heavy metal stress was studied in *D. cespitosa* plants [35] and the Ni hyper-accumulator plant *Alyssum murale* [68]. In summary, *D. armatus* is sensitive to cytokinin treatment, and such treatment is potentially useful in increasing phytoextraction efficiency by inducing metal detoxification.

### 3.7. Phytohormone Content

The presence of phytohormones in *D. armatus* cells in control cultures was confirmed (Figure S10). The application of Cd resulted in a decrease in the content of free forms and ribosides of cytokinins in *D. armatus* cells (Table 3).

The lower levels of biologically active forms of endogenous cytokinins, including a 46.5% reduction in *t*Z, 30.2% in cZ, 35.3% in DHZ, and 38.3% in iP contents, may be attributed to their glycosylation, a type of conjugation process occurring either on the purine ring (*N*-glycosylation at C7 and/or C9) or on the side chain (*O*-glycosylation). It is likely that Cd stimulates this conjugation process, resulting in a 41.0–76.1% increase in *N*-glucosides and a 19.5–63.9% increase in *O*-glucosides of cytokinins, contributing to the downregulation of the active pool of cytokinin free bases. Furthermore, decreases in the contents of auxins, such as IAA, IBA, and PAA, by 35.4%, 61.1%, and 38.9%, respectively, were observed in response to Cd. The synthesis of GA_3_ was inhibited by 53.2% at 72 h of cultivation. The levels of the following brassinosteroids: BL, epiBL, CS, epiCS, CT, HBL, TY, and 6dTY, were reported to decrease in Cd-treated algal cells. Conversely, an increase in the ABA level by 74.8% at 72 h of cultivation was noted, indicating that Cd disrupts homeostasis in algal cells.

Our results suggest that the inhibition in algal growth could be related to decreased levels of auxins, brassinosteroids, biologically active free bases of cytokinins, and gibberellin, which are essential phytohormones regulating cell division. Similar effects have been observed in vascular plants and microalgae exposed to various metals, demonstrating the association between growth suppression and higher toxicity symptoms with increased ABA content and conjugated forms of cytokinins. ABA is known to downregulate plant growth and cell division while upregulating stress responses and tolerance to unfavorable environmental conditions [13,14,24,25,26,27].

The application of exogenous cytokinin (*t*Z) may counteract the inhibitory effect of Cd on *D. armatus* by influencing the endogenous level of ABA, auxins, brassinosteroids, cytokinins, and gibberellin, key phytohormones responsible for cell-cycle progression and stress tolerance mechanisms. Our results indicate that exogenous *t*Z moderated the endogenous contents of different cytokinin forms in algal cultures exposed to Cd stress, leading to an increase in the level of endogenous cytokinin free bases and ribosides. This suggests that *t*Z may reverse Cd toxicity through the stimulation of intracellular auxin levels, contributing to higher tolerance and survival potential in polluted waters.

Exogenously applied *t*Z also increased the content of gibberellin (GA_3_), enhancing algal tolerance to heavy metal presence and supporting the gibberellin-mediated alleviation of metal stress through various mechanisms. The results are also in agreement with observations on alga *E. gracilis*, showing that exogenous *t*Z enabled higher Pb and Cd uptake efficiency and alleviated metal toxicity through the regulation of the levels of endogenous isoprenoid cytokinins and gibberellins [13,14,24,25,26,27]. Furthermore, *t*Z treatment resulted in an increased level of brassinosteroids, suggesting that these phytohormones play a crucial role in stimulating algal growth and metabolism under Cd stress [13,14,24,25,26,27].

Additionally, an increase in ABA content was detected in cells treated with *t*Z + Cd, although its level was lower compared to cultures treated with Cd alone. This indicates that cytokinin may exert an antagonistic effect on ABA synthesis, contributing to an ameliorative effect on algal growth and tolerance under metal stress [13,14,24,25,26,27].

**Figure 8:**
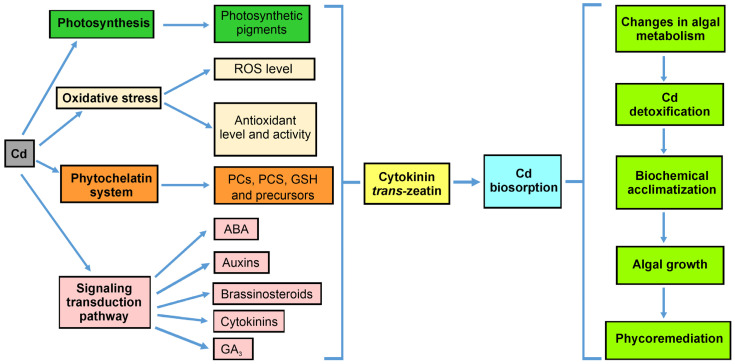
The effect of cytokinin *trans-zeatin* on *D. armatus’* biochemical response, protective mechanisms, and phycoremediation approach under Cd stress.

In summary, our hypothesis that *t*Z is a significant molecule for algal acclimation to metal stress, influencing hormonal homeostasis, has been confirmed. Cytokinin can counteract Cd phytotoxicity by stimulating the synthesis of different phytohormones involved in algal adaptations to stressful conditions. Hormonal crosstalk plays a key role in heavy metal tolerance, involving the activation of PC biosynthesis, stimulation of the antioxidant system, and protection of the photosynthetic apparatus. The interplay between exogenous cytokinin and various endogenous plant hormones under Cd stress may be crucial for utilizing these organisms in the phycoremediation of heavy metals from polluted aquatic environments (Figure 8).

## 4. Conclusions

This study demonstrated that cytokinin is a regulator of *D. armatus* growth, metal accumulation capacity, and acclimation strategies, including PC biosynthesis, enhanced phytohormone accumulation, regulation of hormonal crosstalk, effects on the photosynthetic apparatus, and antioxidant response. These mechanisms collectively improve metal uptake efficiency and algal tolerance to polluted aquatic environments. Our observations of *D. armatus* suggested that *t*Z enhanced the alga’s ability to counteract Cd phytotoxicity. The study also confirmed the additional crosstalk among various hormones (i.e., ABA, auxins, brassinosteroids, cytokinins, and GA_3_) in regulating growth, synthesis of photosynthetic pigments, antioxidant system activity, and PC production under metal stress. Thus, this cytokinin plays a role in regulating the metal bioremediation process and in sourcing valuable organic compounds present in microalgal cells.

## Figures and Tables

**Table 1:** The effect of exogenous cytokinin *trans*-zeatin (*t*Z) on the level of cadmium (Cd) in *D. armatus* during 96 h of cultivation in relation to the control. Treatments with at least one letter the same were not significantly different according to Tukey’s post-hoc test.

Cd Content (amol cell^−1^)				
Treatments	Time of Culture			
	24 h	48 h	72 h	96 h
Control	0	0	0	0
10 µM Cd	76.25 ± 2.002 ^h^	89.14 ± 1.237 ^f^	112.74 ± 1.307 ^d^	121.03 ± 1.884 ^c^
1 µM *t*Z	0	0	0	0
10 µM Cd + 1 µM *t*Z	83.61 ± 1.148 ^g^	94.77 ± 0.941 ^e^	138.32 ± 3.115 ^b^	145.16 ± 3.551 ^a^

**Table 2:** The effect of exogenous cytokinin *trans*-zeatin (*t*Z) and cadmium (Cd) on carotenoids’ contents in *D. armatus* cells during 96 h of cultivation in relation to the control. Treatments with at least one letter the same were not significantly different according to Tukey’s post-hoc test.

Carotene and Xanthophyll Contents (amol cell^−1^)					
Pigments	Treatments	Time of Culture			
		24 h	48 h	72 h	96 h
*α*-Carotene	Control	2.09 ± 0.012 ^h^	2.11 ± 0.101 ^h^	2.56 ± 0.011 ^g^	2.67 ± 0.014 ^g^
	10 µM Cd	2.13 ± 0.023 ^h^	2.55 ± 0.055 ^g^	2.68 ± 0.004 ^g^	3.09 ± 0.021 ^e^
	1 µM *t*Z	2.85 ± 0.041 ^f^	2.87 ± 0.016 ^f^	3.29 ± 0.033 ^d^	3.31 ± 0.036 ^d^
	10 µM Cd + 1 µM *t*Z	2.97 ± 0.043 ^e^	3.48 ± 0.038 ^c^	4.44 ± 0.031 ^a^	4.26 ± 0.010 ^b^
*β*-Carotene	Control	4.12 ± 0.064 ^f^	4.08 ± 0.033 ^f^	4.52 ± 0.032 ^e^	4.66 ± 0.088 ^e^
	10 µM Cd	4.51 ± 0.030 ^e^	4.92 ± 0.075 ^d^	5.17 ± 0.009 ^d^	5.22 ± 0.025 ^d^
	1 µM *t*Z	4.99 ± 0.022 ^d^	4.95 ± 0.019 ^d^	5.78 ± 0.018 ^c^	5.31 ± 0.012 ^d^
	10 µM Cd + 1 µM *t*Z	5.22 ± 0.009 ^d^	5.68 ± 0.099 ^c^	6.99 ± 0.033 ^b^	7.19 ± 0.093 ^a^
Antheraxanthin	Control	4.37 ± 0.046 ^g^	4.52 ± 0.084 ^f^	4.58 ± 0.009 ^f^	4.71 ± 0.014 ^e^
	10 µM Cd	4.45 ± 0.006 ^f^	4.72 ± 0.005 ^e^	4.88 ± 0.016 ^e^	4.83 ± 0.066 ^e^
	1 µM *t*Z	4.46 ± 0.022 ^f^	4.63 ± 0.077 ^e^	4.77 ± 0.044 ^e^	4.69 ± 0.021 ^e^
	10 µM Cd + 1 µM *t*Z	4.99 ± 0.077 ^d^	5.54 ± 0.093 ^c^	7.25 ± 0.064 ^a^	6.63 ± 0.026 ^b^
Violaxanthin	Control	3.78 ± 0.033 ^f^	4.05 ± 0.014 ^e^	4.23 ± 0.067 ^d^	4.19 ± 0.068 ^e^
	10 µM Cd	4.11 ± 0.092 ^e^	4.32 ± 0.021 ^d^	4.66 ± 0.011 ^d^	4.47 ± 0.019 ^d^
	1 µM *t*Z	3.97 ± 0.004 ^f^	4.38 ± 0.015 ^d^	5.45 ± 0.020 ^c^	5.11 ± 0.066 ^c^
	10 µM Cd + 1 µM *t*Z	4.39 ± 0.081 ^d^	5.64 ± 0.085 ^c^	6.93 ± 0.095 ^a^	6.75 ± 0.037 ^b^
Zeaxanthin	Control	10.38 ± 0.921 ^e^	10.46 ± 0.083 ^e^	11.25 ± 0.712 ^d^	11.72 ± 0.997 ^d^
	10 µM Cd	11.16 ± 0.893 ^d^	12.67 ± 0.285 ^d^	12.59 ± 0.805 ^d^	12.31 ± 0.831 ^d^
	1 µM *t*Z	10.48 ± 0.755 ^d^	11.09 ± 0.843 ^d^	12.12 ± 0.722 ^d^	10.75 ± 0.104 ^d^
	10 µM Cd + 1 µM *t*Z	11.65 ± 0.011 ^d^	13.73 ± 0.306 ^c^	14.84 ± 0.101 ^a^	14.21 ± 0.399 ^b^

**Table 3:** The effect of exogenous cytokinin *trans*-zeatin (*t*Z) and cadmium (Cd) on phytohormone contents in *D. armatus* cells at 72 h of cultivation in relation to the control. Treatments with at least one letter the same were not significantly different according to Tukey’s post-hoc test.

Phytohormone Content (fmol cell^−1^)	Treatments			
	Control	10 µM Cd	1 µM *t*Z	10 µM Cd + 1 µM *t*Z
**Cytokinins**				
*trans*-Zeatin (*t*Z)	0.421 ± 0.011 ^c^	0.225 ± 0.015 ^d^	0.865 ± 0.055 ^a^	0.781 ± 0.033 ^b^
*trans*-Zeatin-Riboside (*t*ZR)	0.112 ± 0.015 ^c^	0.082 ± 0.023 ^d^	0.203 ± 0.019 ^a^	0.196 ± 0.008 ^b^
*trans*-Zeatin-9-Glucoside (*t*Z9G)	0.174 ± 0.028 ^c^	0.301 ± 0.101 ^a^	0.292 ± 0.071 ^b^	0.198 ± 0.017 ^c^
*trans*-Zeatin-7-Glucoside (*t*Z7G)	0.071 ± 0.016 ^c^	0.125 ± 0.077 ^a^	0.113 ± 0.007 ^b^	0.072 ± 0.005 ^c^
*trans*-Zeatin-*O*-Glucoside (*t*ZOG)	0.783 ± 0.205 ^c^	0.936 ± 0.069 ^a^	0.855 ± 0.059 ^b^	0.724 ± 0.062 ^c^
*trans*-Zeatin-*O*-Glucoside Riboside (*t*ZROG)	0.097 ± 0.008 ^c^	0.135 ± 0.026 ^a^	0.109 ± 0.013 ^b^	0.088 ± 0.011 ^c^
*cis*-Zeatin (*c*Z)	7.012 ± 0.308 ^c^	4.891 ± 0.274 ^d^	8.804 ± 0.933 ^b^	9.552 ± 0.809 ^a^
*cis*-Zeatin-Riboside (cZR)	3.088 ± 0.264 ^b^	4.294 ± 0.286 ^a^	3.227 ± 0.213 ^b^	3.304 ± 0.244 ^b^
*cis*-Zeatin-*O*-Glucoside (*c*ZOG)	0.585 ± 0.089 ^d^	0.926 ± 0.309 ^a^	0.744 ± 0.019 ^b^	0.633 ± 0.043 ^c^
*cis*-Zeatin-*O*-Glucoside Riboside (*c*ZROG)	0.451 ± 0.042 ^c^	0.739 ± 0.034 ^a^	0.592 ± 0.044 ^b^	0.507 ± 0.013 ^b^
Dihydrozeatin (DHZ)	0.883 ± 0.055 ^b^	0.571 ± 0.028 ^c^	0.992 ± 0.026 ^a^	0.974 ± 0.066 ^a^
Dihydrozeatin Riboside (DHZR)	1.325 ± 0.808 ^c^	1.576 ± 0.605 ^a^	1.489 ± 0.621 ^b^	1.486 ± 0.508 ^b^
Dihydrozeatin-9-Glucoside (DHZ9G)	0.113 ± 0.007 ^c^	0.273 ± 0.080 ^a^	0.178 ± 0.010 ^b^	0.119 ± 0.031 ^c^
Dihydrozeatin-7-Glucoside (DHZ7G)	0.085 ± 0.009 ^b^	0.124 ± 0.018 ^a^	0.099 ± 0.006 ^b^	0.068 ± 0.008 ^c^
Dihydrozeatin-*O*-Glucoside (DHZOG)	0.381 ± 0.026 ^c^	0.519 ± 0.022 ^a^	0.394 ± 0.018 ^c^	0.401 ± 0.016 ^b^
*N*^6^-Isopentenyladenine (iP)	1.807 ± 0.103 ^b^	1.115 ± 0.129 ^c^	1.993 ± 0.237 ^b^	2.155 ± 0.115 ^a^
*N*^6^-Isopentenyladenosine (iPR)	1.749 ± 0.664 ^b^	1.127 ± 0.211 ^c^	1.953 ± 0.075 ^b^	2.038 ± 0.808 ^a^
*N*^6^-Isopentenyladenine-7-Glucoside (iP7G)	0.173 ± 0.059 ^b^	0.244 ± 0.007 ^a^	0.196 ± 0.013 ^b^	0.168 ± 0.037 ^b^
**Auxins**				
Indole-3-acetic acid (IAA)	18.045 ± 1.044 ^b^	11.663 ± 0.803 ^c^	23.251 ± 1.569 ^a^	24.023 ± 2.001 ^a^
Indole-3-butyric acid (IBA)	2.801 ± 0.341 ^b^	1.088 ± 0.017 ^c^	2.955 ± 0.903 ^b^	3.177 ± 0.455 ^a^
Phenylacetic acid (PAA)	5.902 ± 0.099 ^c^	3.605 ± 0.081 ^d^	6.736 ± 0.078 ^b^	7.302 ± 0.811 ^a^
**Abscisic acid** (ABA)	6.318 ± 0.312 ^c^	11.043 ± 0.668 ^a^	5.329 ± 0.408 ^d^	7.034 ± 0.552 ^b^
**Gibberellin** (GA_3_)	1.755 ± 0.308 ^b^	0.822 ± 0.055 ^c^	1.968 ± 0.117 ^b^	2.113 ± 0.081 ^a^
**Brassinosteroids**				
Brassinolide (BL)	2.535 ± 0.088 ^b^	1.604 ± 0.029 ^c^	2.877 ± 0.047 ^a^	2.803 ± 0.048 ^a^
Epibrassinolide (epiBL)	0.671 ± 0.016 ^a^	0.358 ± 0.014 ^b^	0.711 ± 0.085 ^a^	0.739 ± 0.111 ^a^
Castasterone (CS)	3.466 ± 0.052 ^b^	1.684 ± 0.266 ^c^	4.199 ± 0.609 ^b^	5.231 ± 0.446 ^a^
24-Epicastasterone (epiCS)	0.165 ± 0.027 ^b^	0.122 ± 0.037 ^b^	0.227 ± 0.042 ^a^	0.253 ± 0.039 ^a^
Cathasterone (CT)	3.419 ± 0.087 ^b^	2.015 ± 0.807 ^c^	3.881 ± 0.114 ^a^	4.116 ± 0.258 ^a^
28-Homobrassinolide (HBL)	0.137 ± 0.019 ^b^	0.078 ± 0.021 ^c^	0.188 ± 0.035 ^a^	0.175 ± 0.088 ^a^
Typhasterol (TY)	0.275 ± 0.008 ^b^	0.139 ± 0.018 ^c^	0.281 ± 0.067 ^b^	0.316 ± 0.057 ^a^
6-Deoxytyphasterol (6dTY)	0.195 ± 0.033 ^b^	0.108 ± 0.055 ^c^	0.235 ± 0.031 ^a^	0.204 ± 0.041 ^a^

## Data Availability

Data are contained within the current article.

## Supplementary Materials

The following supporting information can be downloaded at: https://www.mdpi.com/article/10.3390/cells13080686/s1, Figure S1. The effect of cadmium (Cd) and *trans*-zeatin (tZ) on the cell number in relation to control at 24, 48, 72, and 92 hours of culture. Data are the means of four independent experiments ± SD. Treatment with at least one letter the same are not significantly different according to Tukey’s Post-hoc test. Figure S2. The chromatogram of photosynthetic pigments in cultures of *Desmodesmus armatus* at the 72 h of cultivation. Figure S3. The chromatogram of photosynthetic pigments in the cultures of *Desmodesmus armatus* treated with Cd at 72 h of cultivation. Figure S4. The chromatogram of photosynthetic pigments in the cultures of *Desmodesmus armatus* treated with *trans*-zeatin at 72 h of cultivation. Figure S5. The chromatogram of photosynthetic pigments in the cultures of *Desmodesmus armatus* treated with Cd and *trans*-zeatin at 72 h of cultivation. Figure S6. The chromatogram of phytochelatins in the cultures of *Desmodesmus armatus* at 72 h of cultivation. Figure S7. The chromatogram of phytochelatins in the cultures of *Desmodesmus armatus* treated with Cd at 72 h of cultivation. Figure S8. The chromatogram of phytochelatins in the cultures of *Desmodesmus armatus* treated with *trans*-zeatin at 72 h of cultivation. Figure S9. The chromatogram of phytochelatins in the cultures of *Desmodesmus armatus* treated with Cd and *trans*-zeatin at 72 h of cultivation. Figure S10. The confirmation of the presence of phytohormones in the cultures of *Desmodesmus* armatus at 72 h of cultivation.
