# Supplementary material for: Mitigating Effect of Trans-Zeatin on Cadmium Toxicity in Desmodesmus armatus
Source: Cells. 2024 Apr 15;13(8):686. doi: 10.3390/cells13080686 (PMC11049045; doi:10.3390/cells13080686)
Supplement: Supplementary file 1 [file cells-13-00686-s001.zip › cells-2936898-supplementary.pdf]

cells

Supplementary Materials

Mitigating Effect of *Trans*-Zeatin on Cadmium Toxicity in *Desmodesmus armatus*

Alicja Piotrowska-Niczyporuk [1], Elżbieta Bonda-Ostaszewska [2] and Andrzej Bajguz [1,*]

[1] Department of Biology and Plant Ecology, Faculty of Biology, University of Bialystok, Ciolkowskiego 1J, 15-245 Bialystok, Poland; alicjap@uwb.edu.pl (A.P.-N.)
[2] Department of Evolutionary and Physiological Ecology, Faculty of Biology, University of Bialystok, Ciolkowskiego 1J, 15-245 Bialystok, Poland; elabonda@uwb.edu.pl (E.B.-O.)
* Correspondence: abajguz@uwb.edu.pl; Tel.: +48-85-7388361 (A.B.)

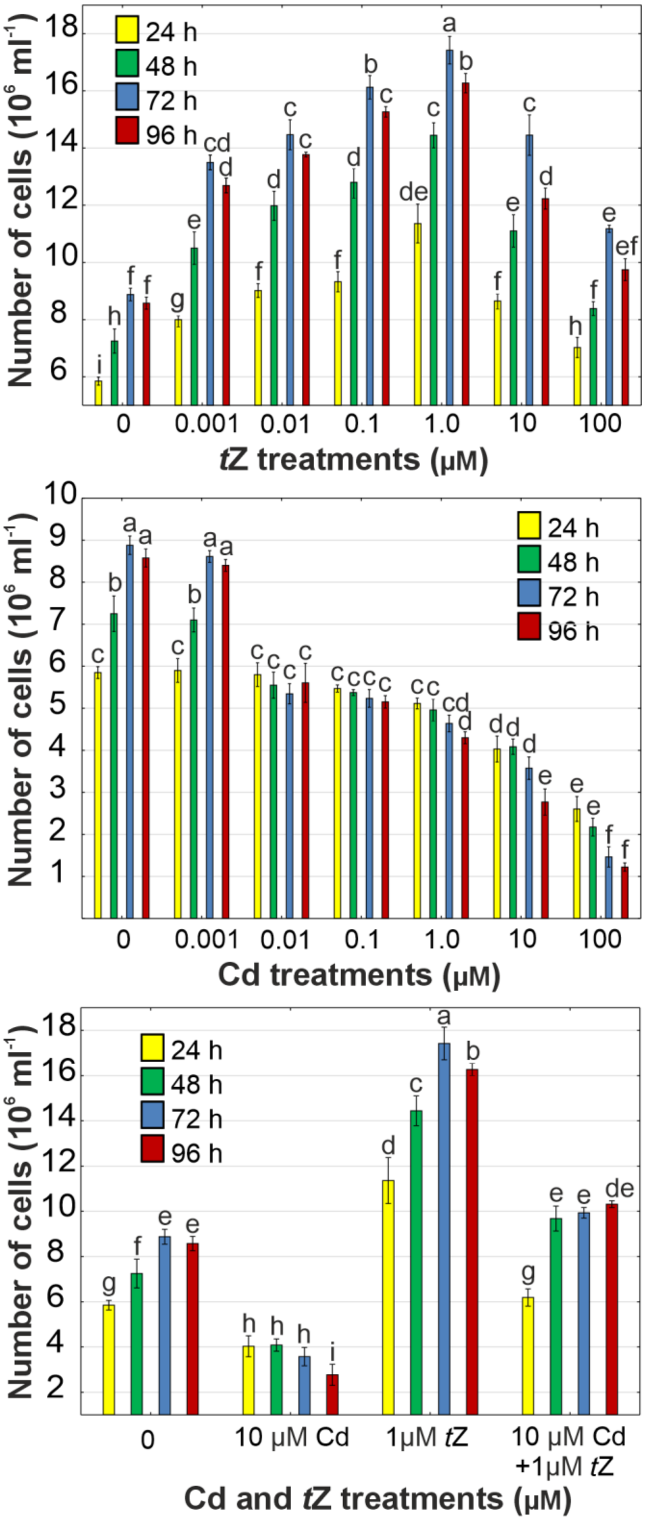

Figure S1. The effect of cadmium (Cd) and *trans*-zeatin (*t*Z) on the cell number in relation to control at 24, 48, 72, and 92 hours of culture. Data are the means of four independent experiments ± SD. Treatment with at least one letter the same are not significantly different according to Tukey's Post-hoc test

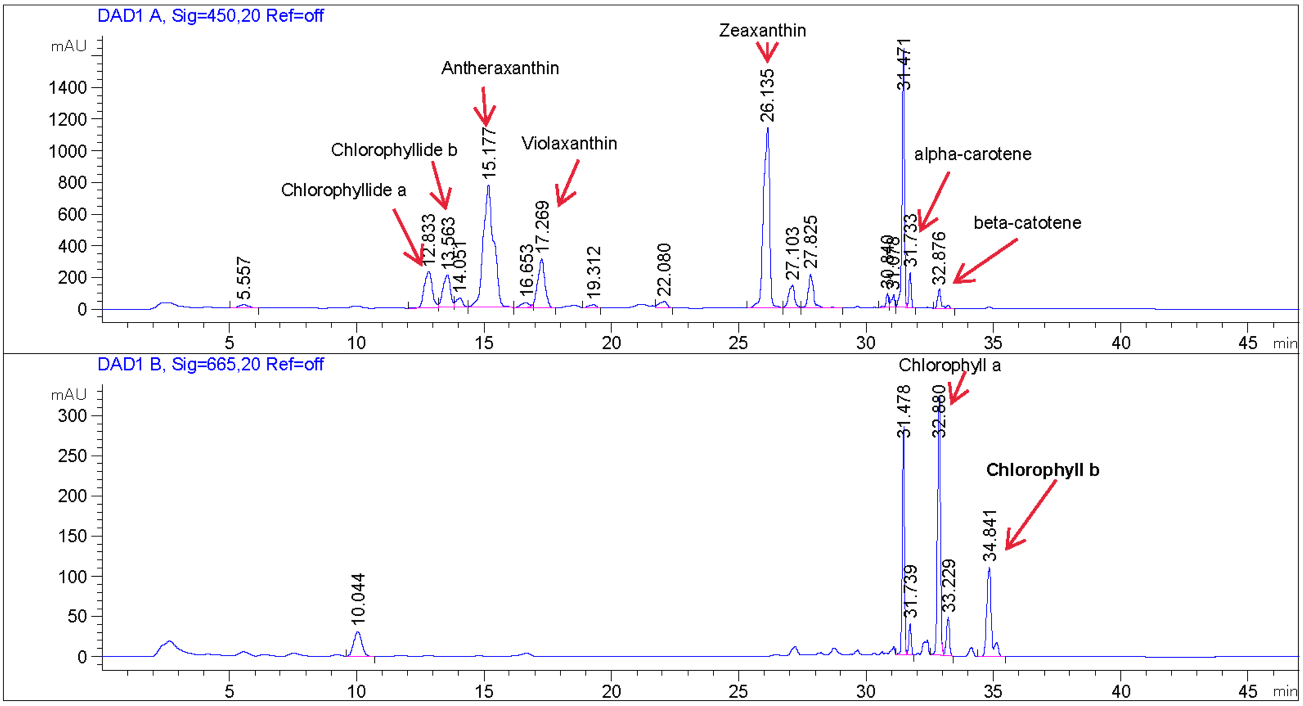

Figure S2. The chromatogram of photosynthetic pigments in cultures of *Desmodesmus armatus* at the 72 h of cultivation

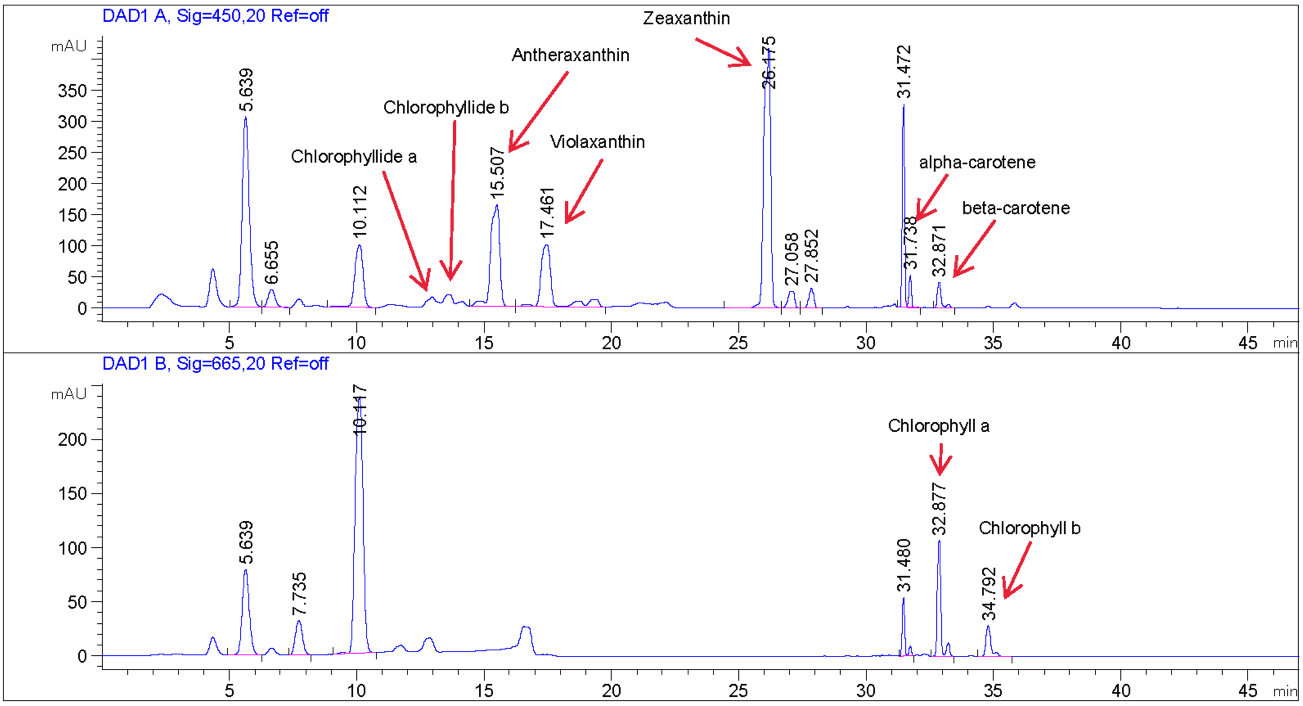

Figure S3. The chromatogram of photosynthetic pigments in the cultures of *Desmodesmus armatus* treated with Cd at 72 h of cultivation

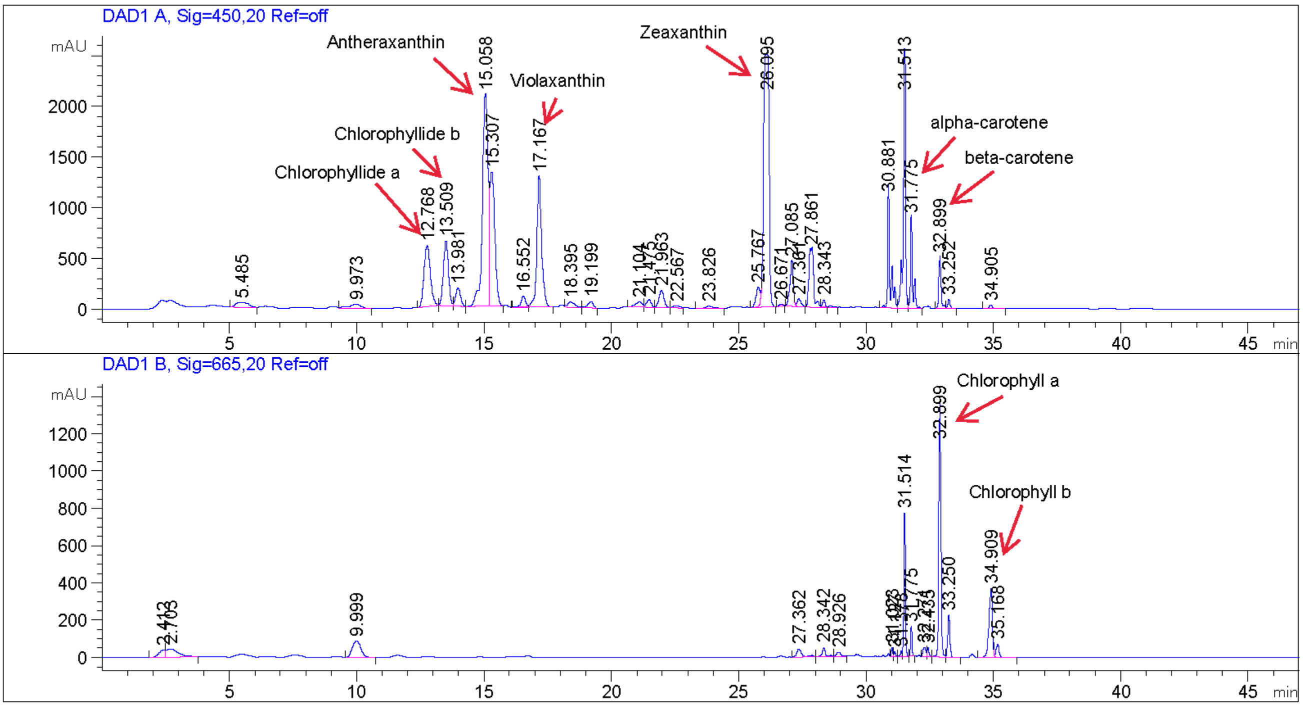

Figure S4. The chromatogram of photosynthetic pigments in the cultures of *Desmodesmus armatus* treated with *trans*-zeatin at 72 h of cultivation

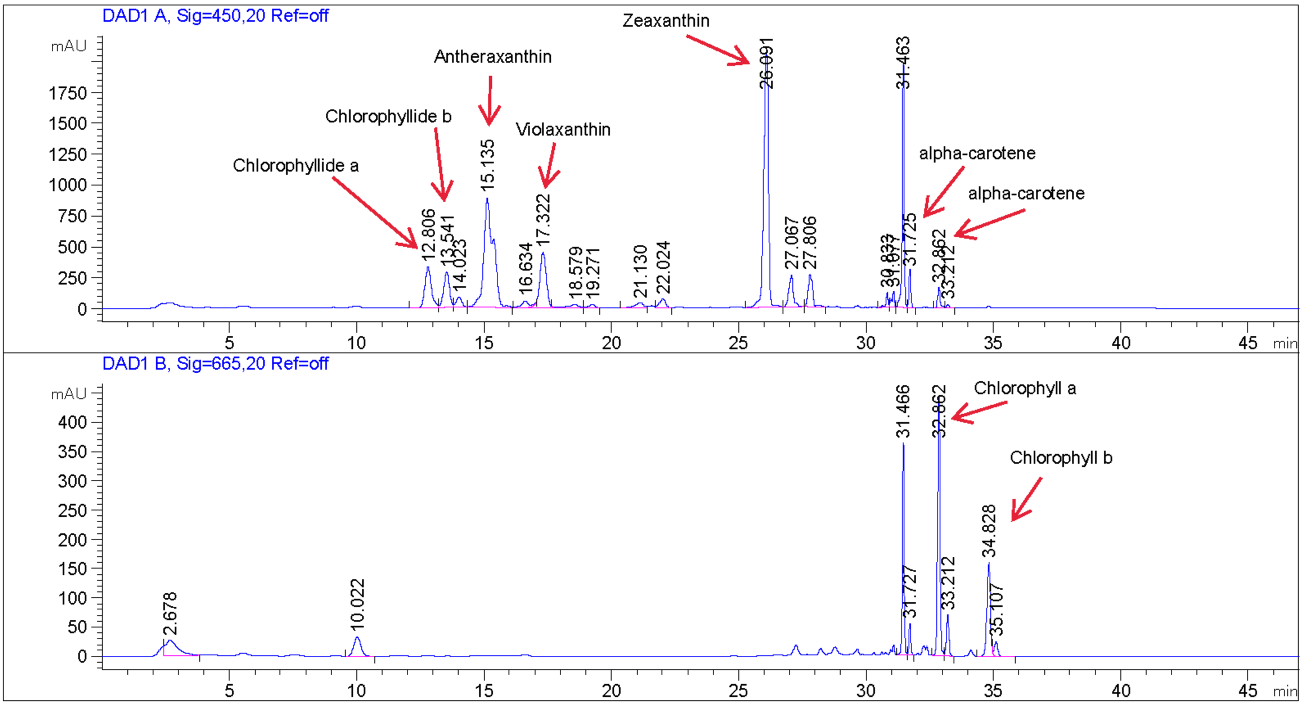

Figure S5. The chromatogram of photosynthetic pigments in the cultures of *Desmodesmus armatus* treated with Cd and *trans*-zeatin at 72 h of cultivation

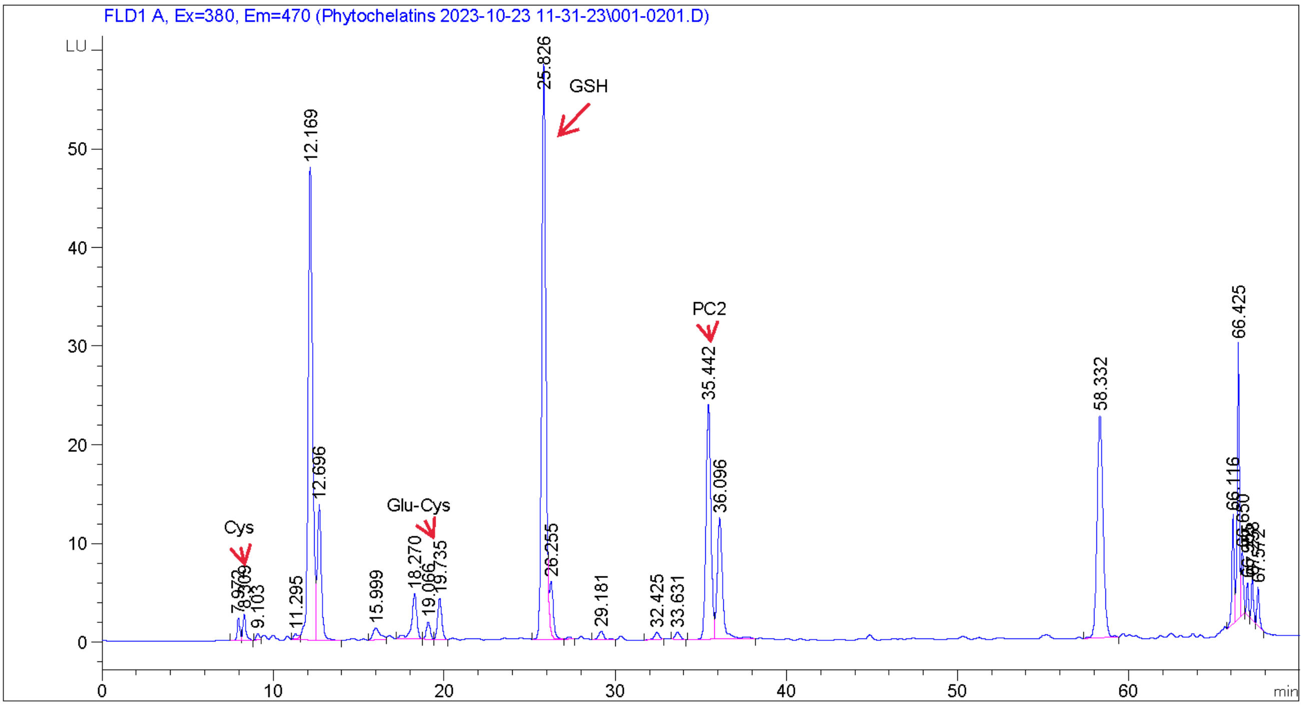

Figure S6. The chromatogram of phytochelatins in the cultures of *Desmodesmus armatus* at 72 h of cultivation

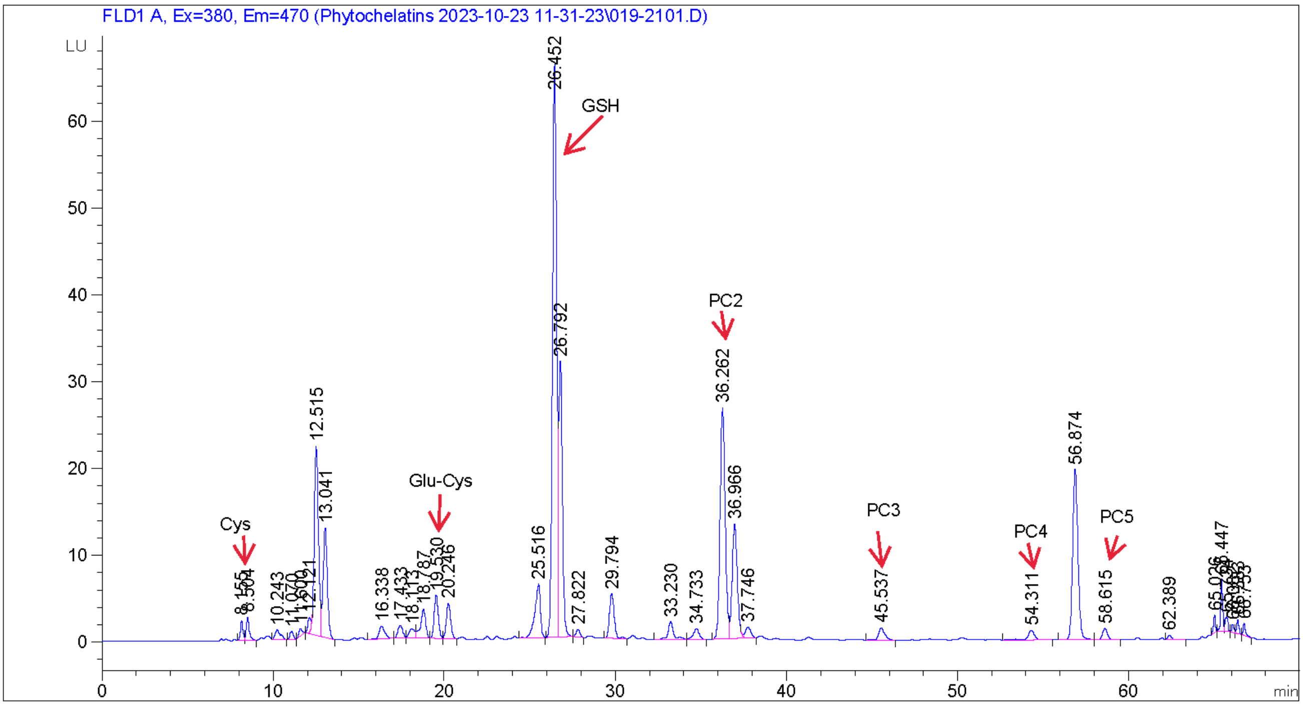

Figure S7. The chromatogram of phytochelatins in the cultures of *Desmodesmus armatus* treated with Cd at 72 h of cultivation

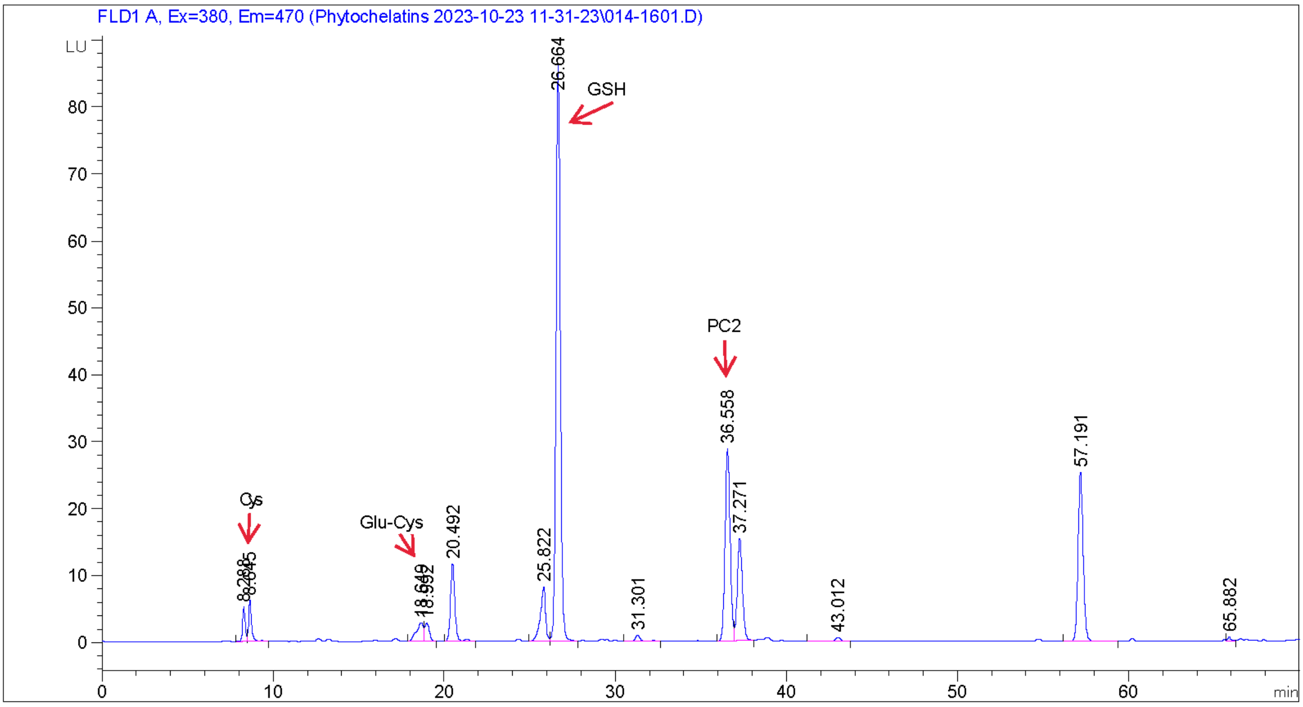

Figure S8. The chromatogram of phytochelatins in the cultures of *Desmodesmus armatus* treated with *trans*-zeatin at 72 h of cultivation

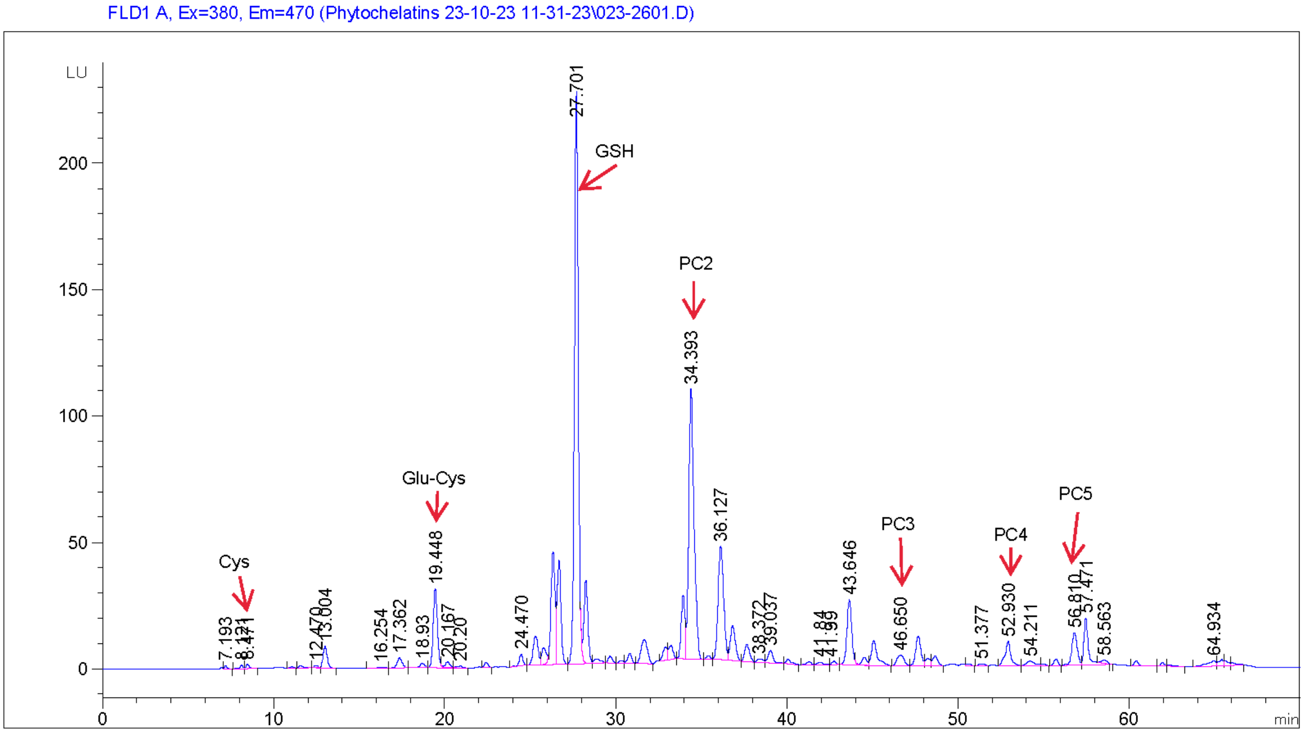

Figure S9. The chromatogram of phytochelatins in the cultures of Desmodesmus armatus treated with Cd and trans-zeatin at 72 h of cultivation

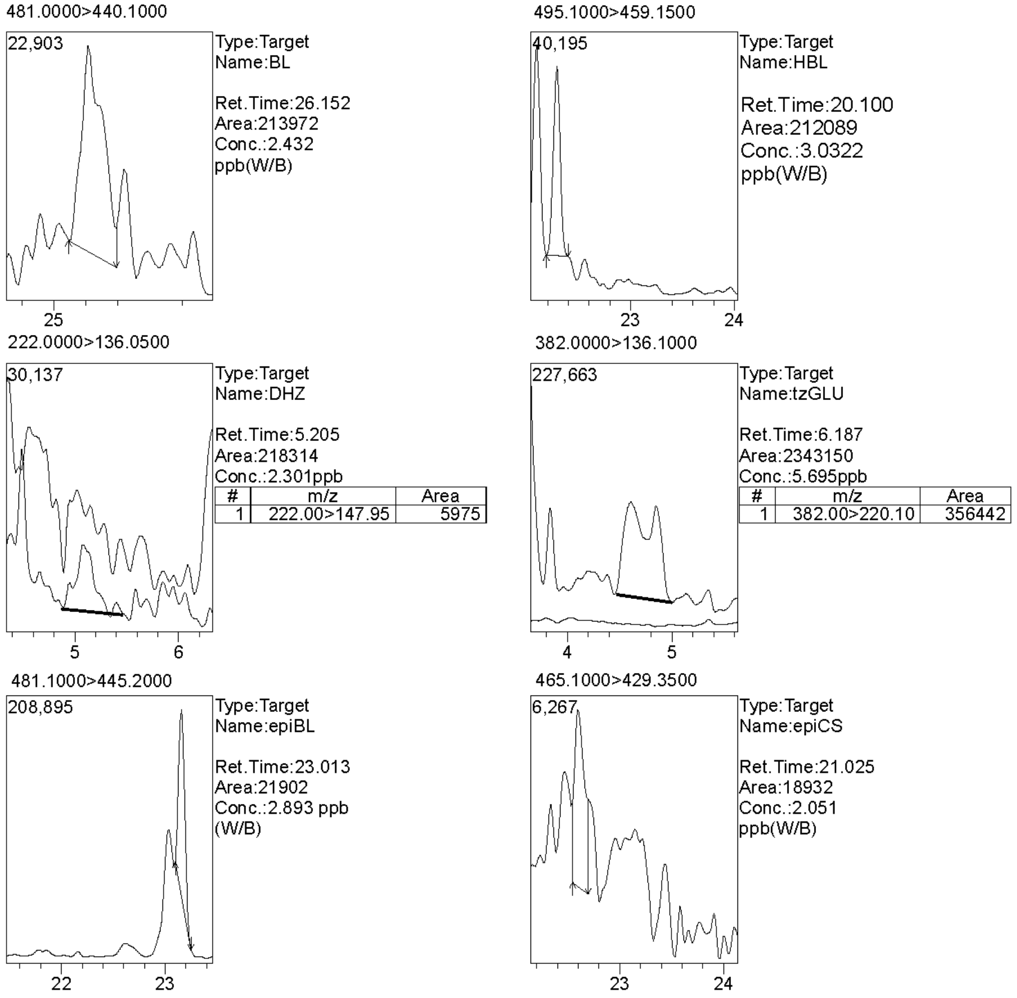

Figure S10. The confirmation of the presence of phytohormones in the cultures of *Desmodesmus armatus* at 72 h of cultivation

220.0000>136.1500

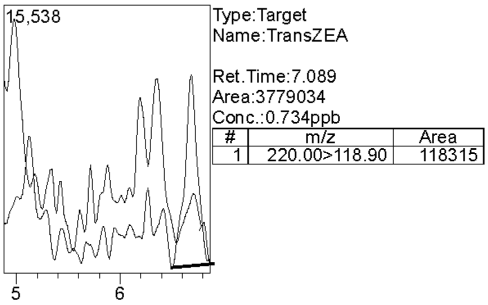

Type:Target
Name:TransZEA

Ret.Time:7.089
Area:3779034
Conc.:0.734ppb

#	m/z	Area
1	220.00>118.90	118315

175.9000>130.3000

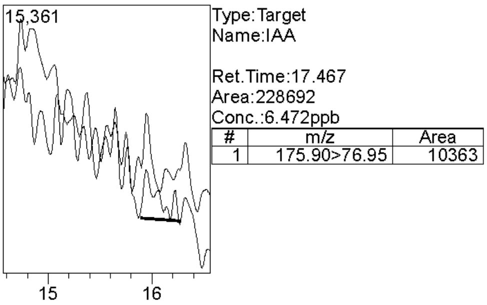

Type:Target
Name:IAA

Ret.Time:17.467
Area:228692
Conc.:6.472ppb

#	m/z	Area
1	175.90>76.95	10363

189.9000>130.2500

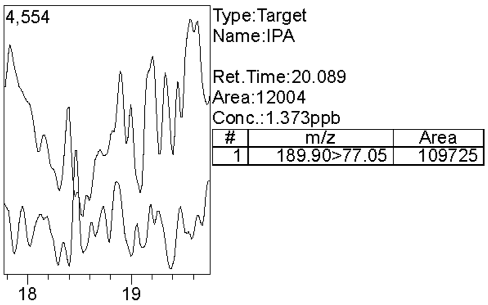

Type:Target
Name:IPA

Ret.Time:20.089
Area:12004
Conc.:1.373ppb

#	m/z	Area
1	189.90>77.05	109725

345.1000>239.0000

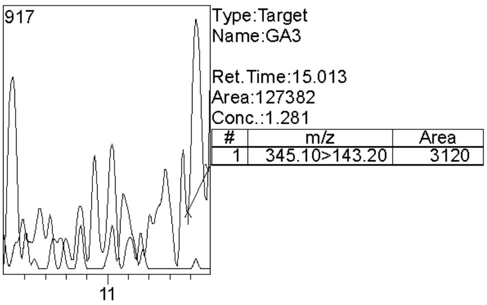

Type:Target
Name:GA3

Ret.Time:15.013
Area:127382
Conc.:1.281

#	m/z	Area
1	345.10>143.20	3120

351.9000>135.9500

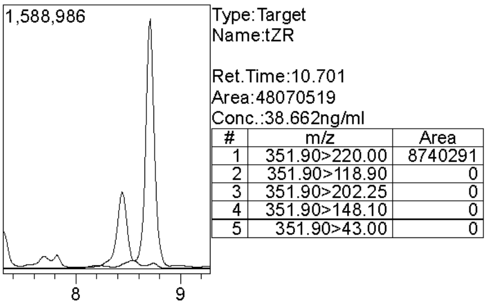

Type:Target
Name:tZR

Ret.Time:10.701
Area:48070519
Conc.:38.662ng/ml

#	m/z	Area
1	351.90>220.00	8740291
2	351.90>118.90	0
3	351.90>202.25	0
4	351.90>148.10	0
5	351.90>43.00	0

381.9000>136.0000

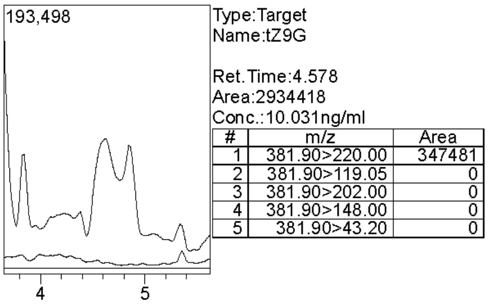

Type:Target
Name:tZ9G

Ret.Time:4.578
Area:2934418
Conc.:10.031ng/ml

#	m/z	Area
1	381.90>220.00	347481
2	381.90>119.05	0
3	381.90>202.00	0
4	381.90>148.00	0
5	381.90>43.20	0

381.9000>220.2000

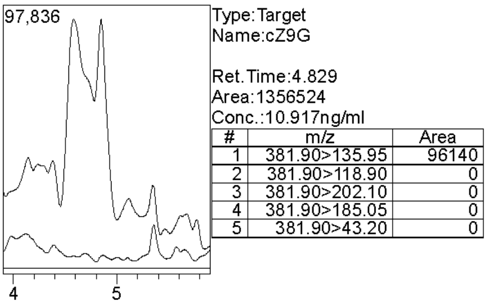

Type:Target
Name:cZ9G

Ret.Time:4.829
Area:1356524
Conc.:10.917ng/ml

#	m/z	Area
1	381.90>135.95	96140
2	381.90>118.90	0
3	381.90>202.10	0
4	381.90>185.05	0
5	381.90>43.20	0

Figure S10. *Continued.*

351.9000>220.0500

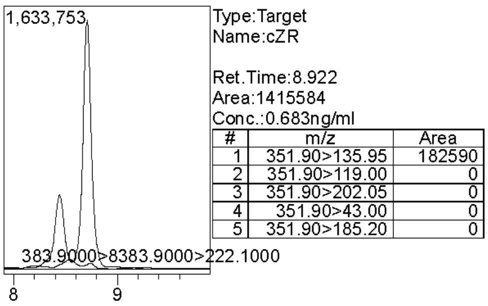

Type:Target
Name:cZR

Ret.Time:8.922
Area:1415584
Conc.:0.683ng/ml

#	m/z	Area
1	351.90>135.95	182590
2	351.90>119.00	0
3	351.90>202.05	0
4	351.90>43.00	0
5	351.90>185.20	0

353.9000>222.0000

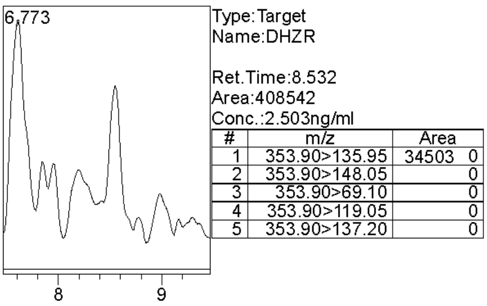

Type:Target
Name:DHZR

Ret.Time:8.532
Area:408542
Conc.:2.503ng/ml

#	m/z	Area
1	353.90>135.95	34503 0
2	353.90>148.05	0
3	353.90>69.10	0
4	353.90>119.05	0
5	353.90>137.20	0

383.9000>137.0500

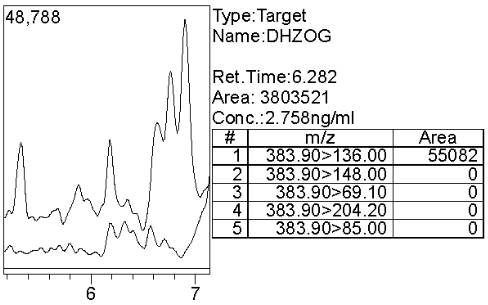

Type:Target
Name:DHZOG

Ret.Time:6.282
Area: 3803521
Conc.:2.758ng/ml

#	m/z	Area
1	383.90>136.00	55082
2	383.90>148.00	0
3	383.90>69.10	0
4	383.90>204.20	0
5	383.90>85.00	0

383.9000>222.1000

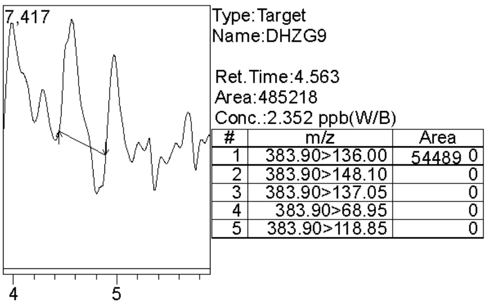

Type:Target
Name:DHZG9

Ret.Time:4.563
Area:485218
Conc.:2.352 ppb(W/B)

#	m/z	Area
1	383.90>136.00	54489 0
2	383.90>148.10	0
3	383.90>137.05	0
4	383.90>68.95	0
5	383.90>118.85	0

383.9000>222.2000

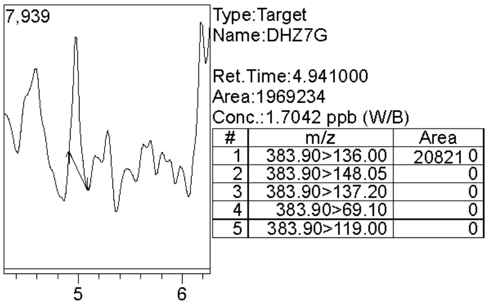

Type:Target
Name:DHZ7G

Ret.Time:4.941000
Area:1969234
Conc.:1.7042 ppb (W/B)

#	m/z	Area
1	383.90>136.00	20821 0
2	383.90>148.05	0
3	383.90>137.20	0
4	383.90>69.10	0
5	383.90>119.00	0

203.9000>135.9500

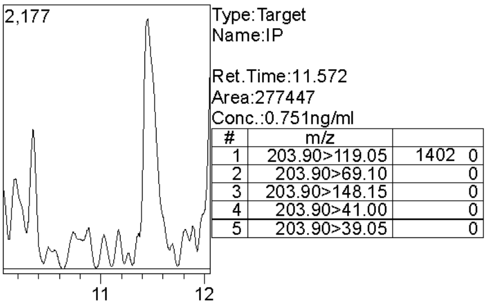

Type:Target
Name:IP

Ret.Time:11.572
Area:277447
Conc.:0.751ng/ml

#	m/z	
1	203.90>119.05	1402 0
2	203.90>69.10	0
3	203.90>148.15	0
4	203.90>41.00	0
5	203.90>39.05	0

335.9000>136.0000

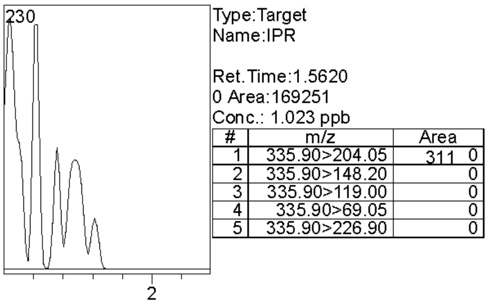

Type:Target
Name:IPR

Ret.Time:1.5620
0 Area:169251
Conc.: 1.023 ppb

#	m/z	Area
1	335.90>204.05	311 0
2	335.90>148.20	0
3	335.90>119.00	0
4	335.90>69.05	0
5	335.90>226.90	0

365.8000>136.0500

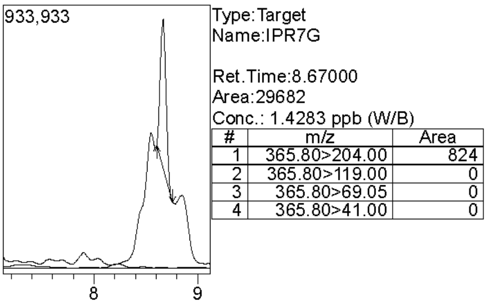

Type:Target
Name:IPR7G

Ret.Time:8.67000
Area:29682
Conc.: 1.4283 ppb (W/B)

#	m/z	Area
1	365.80>204.00	824
2	365.80>119.00	0
3	365.80>69.05	0
4	365.80>41.00	0

Figure S10. *Continued.*

203.8000>186.0000

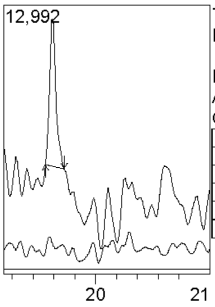

Type:Target
Name:IBA

Ret.Time:19.631
Area:23908
Conc.:1.251 ppb (W/B)

#	m/z	Area
1	203.80>130.00	5103
2	203.80>144.10	0
3	203.80>117.00	0
4	203.80>168.00	0
5	203.80>103.05	0

467.1000>431.1000

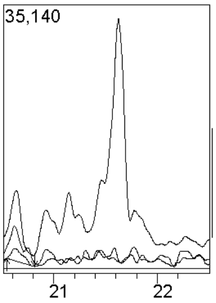

Type:Target
Name:NorBL

Ret.Time:21.361 Area:30314
Conc.:0.894 ppb (W/B)

#	m/z	Area
1	467.10>81.20	2782
2	467.10>413.15	0
3	467.10>315.10	0
4	467.10>95.15	0
5	467.10>313.10	0

434.7000>394.0500

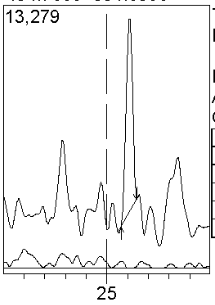

Type:Target
Name:6dTY

Ret.Time:0.000
Area:0
Conc.:N.D.(W/B)

#	m/z	Area
1	434.70>352.75	0
2	434.70>312.25	0
3	434.70>373.00	0
4	434.70>416.50	0
5	434.70>388.90	0

432.7000>365.1500

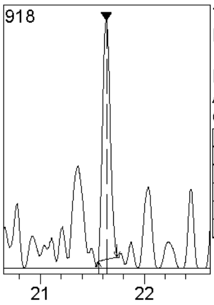

Type:Target
Name:CT

Ret.Time:21.634
Area:4233
Conc.:4.194ng/ml

#	m/z	Area
1	432.70>391.85	0
2	432.70>351.00	0
3	432.70>368.60	0
4	432.70>415.05	0
5	432.70>309.10	0

Figure S10. *Continued.*

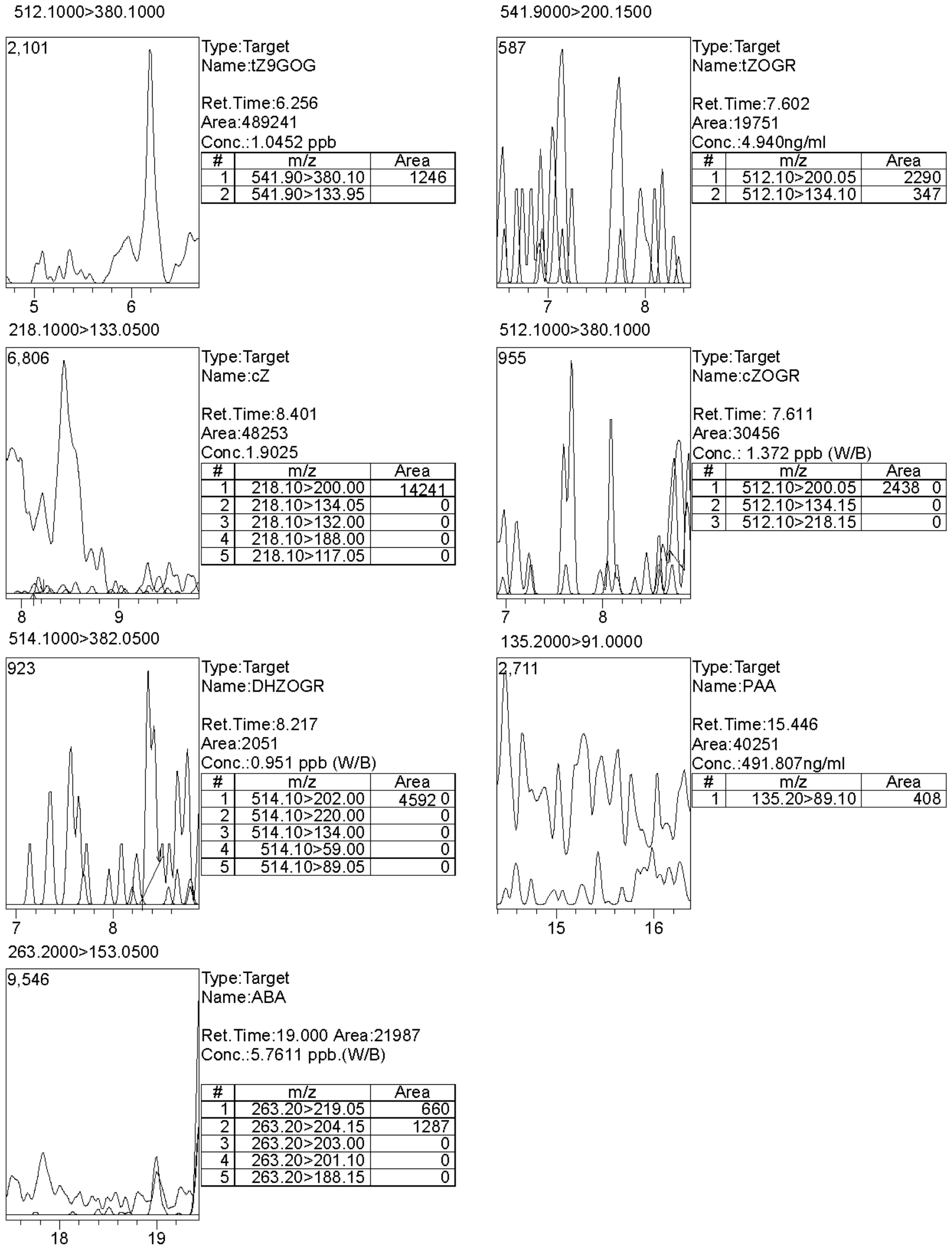

Figure S10. *Continued.*
